# Mettl1-mediated m^7^G modification of Fgfr2 regulates osteogenic and chondrogenic differentiation of mesenchymal stem cells

**DOI:** 10.7150/ijbs.114889

**Published:** 2025-09-03

**Authors:** Quanfeng Li, Yunhui Zhang, Pengfei Ji, Yibin Zhang, Jianan Jiang, Jiahao Jin, Zihao Yuan, Guangqi Tian, Mingxi Cai, Pei Feng, Yanfeng Wu, Wenjie Liu, Peng Wang

**Affiliations:** 1 Department of Traditional Chinese Medicine, Sun Yat-Sen Memorial Hospital, Sun Yat-sen University, Guangzhou, 510120, China.; 2 Department of Orthopedics, The Eighth Affiliated Hospital, Sun Yat-sen University, Shenzhen, 518033, China.; 3 Guangdong Provincial Clinical Research Center for Orthopedic Diseases, The Eighth Affiliated Hospital, Sun Yat-sen University, Shenzhen, 518033, China.; 4 Center for Biotherapy, The Eighth Affiliated Hospital, Sun Yat-sen University, Shenzhen, 518033, China.

## Abstract

N7-methylguanosine (m^7^G) is a prevalent RNA modification and plays fundamental roles in embryonic stem cell self-renewal and differentiation. However, its specific contributions to mesenchymal stem cell differentiation during skeletal development remain poorly understood. In this study, we demonstrate that specific deletion of the m^7^G methyltransferase Mettl1 in mesenchymal lineage cells causes severe bone development defects, manifesting as dramatic limb shortening at birth. The absence of Mettl1 in mesenchymal stem cells significantly hinders osteoblast and chondrocyte differentiation. Integrative analyses of single-cell RNA-sequencing and m^7^G-MeRIP sequencing demonstrate that Mettl1 ablation disrupts m^7^G modifications of Fgfr2, resulting in reduced its mRNA stability. Fgfr2 downregulation impairs the PI3K-AKT and MAPK signaling pathways, which decreases Sp1 phosphorylation and promotes its ubiquitin-mediated degradation, ultimately leading to reduced transcription of Col1a1 and Col2a1. Pharmacological reactivation of Fgfr2 signaling rescues the defects caused by Mettl1 deletion. Our findings highlight the critical role of Mettl1-mediated m^7^G modification in regulating osteogenic and chondrogenic differentiation of mesenchymal stem cells during bone development and provide new insights into the regulatory mechanisms of RNA modifications in skeletal biology.

## Introduction

Skeletogenesis commences with the migration of mesenchymal stem cells from diverse lineages to prospective bone sites 1, 2. At these sites, mesenchymal stem cells (MSCs) aggregate into dense clusters, shaping the developing bone. Within these clusters, mesenchymal stem cells differentiate mainly into two lineages: chondrocytes, which form cartilage models (endochondral ossification), and osteoblasts, which directly create bone (intramembranous ossification) 3-5. In the process of bone synthesis, fibroblast growth factors (FGFs)/ fibroblast growth factor receptors (FGFR 1-5) signaling is a crucial skeletal component to determine cell fate and facilitate bone mineralization through the activation of intracellular signaling pathways, including MAPK, PI3K/AKT, STAT1/p21, JNK, and p38 6. Accumulating evidence has confirmed the essential involvement of FGFRs in skeletal morphogenesis, with mutations in Fgfr2 known to cause skeletal abnormalities such as craniosynostosis and dwarfism by disrupting signal transduction 7. However, the precise mechanisms by which FGF/FGFRs signaling orchestrates bone development across diverse cellular populations and during distinct developmental phases remain complex and require further study.

N^7^-methylguanosine (m^7^G) is a prevalent RNA modification that regulates various RNA functions across tRNA, rRNA, and mRNA 8. The m^7^G modification at position 46 of tRNAs (m^7^G_46_) protects tRNA from degradation and enhances protein translation 9, 10. Similarly, the m^7^G mark at position 1639 (m^7^G_1639_) of 18S rRNA is involved in the processing of pre-18S rRNA and maturation of the 40S ribosomal subunit 11, 12. The m^7^G cap at the 5' end of mRNA is crucial for mRNA stability, facilitating splicing, efficient translation, and other regulatory functions 13-16. In mammals, the m^7^G modification is catalyzed by the Mettl1-Wdr4 complex, with Mettl1 acting as the methyltransferase and Wdr4 facilitating the complex to bind to target RNA 17. The absence of Mettl1 and/or Wdr4 hinders nervous system development and the differentiation of mouse embryonic stem cells 18, 19. In humans, abnormal m^7^G modification due to Wdr4 mutations are linked to primordial dwarfism, highlighting the role of m^7^G in both physiological and pathological processes 20. However, the role of m^7^G modification in skeletal biology remains largely unexplored.

In this study, we demonstrate that conditional knockout of *Mettl1* in mesenchymal stem cells with *Prrx1-Cre* severely impairs bone formation by inhibiting osteoblast and chondrocyte differentiation. Through single-cell RNA sequencing and m^7^G MeRIP sequencing of mouse femoral tissues, we revealed that Mettl1 deletion disrupted m^7^G modifications of Fgfr2. Mechanistically, Mettl1 deficiency led to inactivation of Fgfr2 signaling, decreased Sp1 phosphorylation, and increased Sp1 ubiquitin-mediated degradation, which ultimately reduced transcription of Col1a1 and Col2a1. Our results emphasize the essential role of Mettl1-mediated m^7^G modification in osteogenic and chondrogenic differentiation of mesenchymal stem cells during bone development and offer new insights into the regulatory mechanisms of RNA modifications in skeletal biology.

## Materials and Methods

### Mice

*Prrx1-cre* mice (C57BL/6) were purchased from the Jackson Laboratory. *Mettl1^flox/-^* (C57BL/6) transgenic mice were purchased from GemPharmatech to construct *Mettl1^flox/flox^Prrx1^cre^* mice. Only the 208-bp DNA bands will be detected in wild-type mice, and only the 313-bp DNA bands will be detected in homozygous *Mettl1^flox/flox^* mice. Both 208-bp and 313-bp DNA bands will be detected in heterozygous mice (*Mettl1^flox/-^*). Offsprings were genotyped by PCR using the following primer sequences:

*Mettl1^flox/flox^*: primer #1 5'-CAAGCCACTGGTCTATGTTCACATC-3' and primer #2 5'-TGCCCAGCATCATTTGGTCTT-3'.

*Prrx1-cre*: primer #3 5'-AGCGATGGATTTCCGTCTCTGG-3' and primer #4 5'-AGCTTGCATGATCTCCGGTATTGAA-3'.

### Skeletal staining

Newborn mice were euthanized using CO_2_. Following the excision of skin and internal organs, the mice were fixed overnight in 95% ethanol and then stained overnight in a solution of 0.015% Alcian blue in 80% ethanol. The specimens were cleaned in 95% ethanol for 3 hours, followed by 24 hours in 2% KOH. The skeletons were then stained overnight in a solution of 0.005% Alizarin Red S in 1% KOH. Finally, the specimens were cleared in 1% KOH until they became fully transparent.

### Histology and immunohistochemical assay

Bone tissues designated for paraffin sectioning were meticulously dissected and fixed overnight at room temperature in 4% paraformaldehyde. Following this, they were decalcified for one week in 14% EDTA (pH 7.2) before embedding in paraffin. Sections were stained using various methods, including H&E, Safranin O/Fast Green, and Masson's stain. The TUNEL assay was performed using the One Step TUNEL Apoptosis Assay Kit (Beyotime, C1086). After deparaffinization and rehydration, sections underwent antigen retrieval by incubation in citrate buffer (10 mM) and microwaving at 750 W for 20 minutes. Once cooled to room temperature, the sections were treated with 3% hydrogen peroxide for 25 minutes, followed by a 30-minute incubation in 5% normal goat serum. The sections were then incubated overnight at 4 °C with anti-Mettl1 (Abcam, ab271063) and anti-collagen I (Abcam, ab34710) antibodies. The next day, secondary antibodies were applied, and color development was achieved using an SP Rabbit & Mouse HRP Kit (CWBIO, CW2069S). Finally, microscopy images were captured.

### Immunofluorescence assay

The collected bone samples underwent fixation, decalcification, rehydration, and antigen retrieval as previously described. After treatment with a 0.5% Triton X-100 solution for 30 minutes, the sections were blocked using 5% normal goat serum. They were then incubated overnight at 4 °C with anti-Ki67 (Proteintech, 27309-1-AP), anti-Aggrecan (Proteintech, 13880-1-AP), or anti-Col10a1 (Proteintech, 26984-1-AP) antibodies. The following day, after washing with PBS, the sections were incubated for one hour with fluorescein-labeled secondary antibodies (Abcam, ab150114 and ab150077). For mounting, DAPI antifade mounting medium (Beyotime, P0131) was utilized. Subsequently, microscopy images were captured.

### Micro‑CT analysis

Micro-CT assays were conducted using the Inveon MM system (Siemens) to evaluate bone structures. Images were acquired with 360 rotational steps, featuring a pixel size of 8.82 µm, a voltage of 80 kV, a current of 500 µA, and an exposure time of 1500 ms. Key parameters, including the bone volume/tissue volume (BV/TV) ratio, trabecular thickness (Tb. Th), trabecular number (Tb. N), cortical thickness (Ct. Th), and trabecular separation (Tb. Sp), were analyzed using Inveon Research Workplace software from Siemens.

### Protein extraction and Western blot assay

The collected bone tissues were pulverized using a grinding vessel in RIPA buffer supplemented with protease and phosphatase inhibitors, maintaining the mixture on ice for 30 minutes. After centrifugation at 14,000 rpm for 10 minutes at 4 °C, the lysates were collected and mixed with 5% SDS loading buffer. The protein lysates were then subjected to SDS-PAGE and transferred to polyvinylidene fluoride membranes (Millipore). The membranes were blocked with 5% nonfat dry milk in TBST (150 mM NaCl, 50 mM Tris-HCl, 0.05% Tween 20) for 1 hour at room temperature, followed by an overnight incubation with primary antibodies at 4 °C. After this, secondary antibodies (Cell Signaling Technology, 7074 and 7076) were applied for 1 hour at room temperature. Protein levels were detected using chemiluminescent reagents (Millipore, WBKLS0500) following the manufacturer's instructions.

### RNA extraction and Quantitative Real-Time PCR (qPCR)

Total RNA was extracted using TRIzol solution (AG, #21102) according to the manufacturer's instructions. The RNA was then reverse transcribed into cDNA using the Evo M-MLV RT Master Mix (AG, #11706) on a PCR amplifier. qPCR was performed using the SYBR Green Premix Pro Taq HS qPCR Kit (AG, #11718). Gene expression was measured with a real-time fluorescence quantitative PCR system (Applied Biosystems, 7500). GAPDH served as the reference gene, and relative gene expression was determined using the 2^-ΔΔCt^ method.

### Single-cell RNA sequencing (Sc-RNA seq)

Eight fresh femur tissues (two from each group of mice at postnatal days 3, 7, 14, and 25) were prepared for scRNA sequencing assays. The femur tissues were enzymatically digested with 1 mg/ml collagenase P for 20 minutes, and the resulting cell suspension was then collected for sequencing. Cell density along with live cell proportion was assessed using a Countess II Automated Cell Counter. Samples with a density of 1000 cells per μl and a live cell percentage greater than 95% were selected for further scRNA sequencing. Briefly, samples were loaded onto a 10X Genomics Chromium single-cell controller to generate single-cell gel beads-in-emulsion (GEMs). The captured cells were lysed, and the released RNA was barcoded through reverse transcription within individual GEMs. A single-cell RNA library was then prepared using the Chromium Single Cell 3′ Library & Gel Bead Kit V3 according to the manufacturer's protocols. After quality control, the cDNA libraries were sequenced on an Illumina NovaSeq 6000 sequencer with a sequencing depth of at least 100,000 reads per cell, employing a paired-end 150 bp (PE150) reading strategy.

### Single-cell RNA data analysis

Sequenced reads were demultiplexed and aligned to the mouse GRCm38/mm10 reference genome using CellRanger (version 7.2.0) with default parameters. The cell barcodes and unique molecular identifiers (UMIs) associated with the aligned reads underwent correction and filtering, leading to the construction of the gene barcode matrix for each sample. Subsequent analyses, including data normalization, dimensionality reduction, and K-means cell clustering, were performed using R (version 4.1.2) with the Seurat package (version 4.1.1). Gene Ontology (GO) and Kyoto Encyclopedia of Genes and Genomes (KEGG) analyses were conducted using DAVID Bioinformatics Resources (version 6.8). Additionally, a single-cell trajectory analysis was built with Monocle (R package), which introduced pseudotime. Genes were filtered based on the following criteria: expressed in more than 10 cells, an average expression value greater than 0.1, and a q-value less than 0.01 across different analyses.

### m^7^G dot blot

Fresh femur tissues were processed for RNA extraction, yielding total RNA, of which 100 ng was spotted onto a nylon membrane (Beyotime, FFN56). The membranes were UV crosslinked and blocked with PBST (PBS with 0.1% Tween 20) containing 5% nonfat milk for 1 hour. Subsequently, the membranes were incubated with m7G methylation antibody (Proteintech, 68302-1-Ig, 1:1000) at 4 °C overnight. After washing with PBST, they were incubated with HRP-conjugated secondary antibodies (Cell Signaling Technology, 7074) for 1 hour at room temperature. Following additional washes with PBST, detection was achieved using Immobilon Western Chemiluminescent HRP Substrate (Millipore WBKLS0500). For comparison, an equal amount of RNA was spotted on a second nylon membrane, subjected to UV crosslinking, and stained with 0.02% methylene blue solution (pH 5.2) for 1 hour. The membranes were then washed with ribonuclease-free water for 2 hours, and results were captured via camera.

### m^7^G -MeRIP sequencing

Total RNA was subjected to immunoprecipitation using the GenSeq® m7G MeRIP Kit (GenSeq Inc.) according to the manufacturer's instructions. Briefly, RNA was decapped with Decapping Enzyme and then randomly fragmented to approximately 200 nt using RNA Fragmentation Reagents. Protein A/G beads were coupled to the m^7^G antibody by rotating at room temperature for 1 hour. The RNA fragments were incubated with the bead-bound antibodies and rotated at 4 °C for 4 hours. The RNA/antibody complexes were then digested with Proteinase K, and the eluted RNA was purified by phenol: chloroform extraction. RNA libraries for immunoprecipitation (IP) and input samples were constructed using the GenSeq® Low Input RNA Library Prep Kit (GenSeq Inc.) following the manufacturer's instructions. Libraries were assessed for quality using the Agilent 2100 Bioanalyzer (Agilent) before sequencing.

### Primary mice MSCs isolation

Fresh femur tissues were prepared for the isolation of primary mice MSCs. The femoral tissue was minced and rinsed with PBS, then filtered through a 70 μm filter membrane. After resuspension, the cells were cultured in DMEM/F12 medium. After 7-10 days, adherent cells, which were identified as MSCs, were distinguished from non-adherent cells, which mainly consisted of blood and immune cells. The supernatant was removed, and the remaining cells were washed with PBS to remove any suspended cells, leaving only the MSCs.

### RNA immunoprecipitation (RIP)

An EZ-Magna RIP™ RNA-Binding Protein Immunoprecipitation Kit (Millipore, 17-701) was utilized according to the manufacturer's instructions. Briefly, total RNA was isolated from bone marrow-derived MSCs and incubated with magnetic beads conjugated to anti-m7G (MBL, RN017M, 1:100), anti-Mettl1 (Abcam, ab271063, 1:100), or negative control IgG (Abcam, ab172730, 1:100). The immunoprecipitated RNAs were purified and extracted. The obtained RNAs and input RNAs were then subjected to qPCR to detect the levels of Fgfr2.

### Lentivirus construction and infection

Cre lentiviruses and overexpression lentiviruses for wild-type Mettl1 (Mettl1 WT), mutant Mettl1 (Mettl1 mut), wild-type Fgfr2 (Fgfr2 WT), and mutant Fgfr2 (Fgfr2 mut) were purchased from Obio (Shanghai, China). Cells were infected with the lentivirus at a multiplicity of infection (MOI) of 20, using polybrene (5 µg/ml). The infection medium was replaced 24 hours post-infection, and the infection efficiency was assessed by qPCR 48 hours later.

### RNA stability assay

MSCs were seeded, and gene interference or overexpression was performed as described previously. Seventy-two hours later, actinomycin D (2 µg/ml) was added to inhibit transcription, and total RNA was extracted at 0, 60, 120, 180, and 240 minutes. The expression of target genes at these time points was assessed by qPCR, and the degradation rate was analyzed.

### Dual‑luciferase reporter assay

The Sp1-binding promoter regions of Col1a1 and Col2a1, along with their complementary antisense sequences, were chemically synthesized and individually inserted into pGL3 expression vectors. 293T cells were sequentially transfected with these recombinant plasmids followed by lentiviral delivery systems to achieve Mettl1 knockout and Fgfr2/Sp1 overexpression. To normalize transfection efficiency, all experimental groups were co-transfected with pRL-TK plasmids containing Renilla luciferase. Dual-luciferase activity was quantified using the Promega Dual-Luciferase Reporter Assay System (E1910, Promega Corporation, MA, USA), with relative luciferase intensity calculated as the ratio of firefly to Renilla luminescence signals.

### Cell function assays of MSCs *in vitro*

Cell proliferation was assessed using the EdU Cell Proliferation Kit (Beyotime, C0075), and images were captured with a fluorescence microscope (Leica-DMi8). After inducing osteogenic and chondrogenic differentiation of MSCs, we stained the cells with Alizarin Red S (Beyotime, C0138) and Alcian Blue staining solutions (Beyotime, C0155M), respectively. The extent of mineralization was quantified by measuring the absorbance of Alizarin Red S staining at 562 nm using a microplate reader. Additionally, the areas stained by Alizarin Red and Alcian Blue were analyzed using ImageJ software to assess the degree of mineralization and cartilage formation.

### Statistical analysis

The study presents data as scatter plots, highlighting means and standard deviations. We conducted statistical analyses using GraphPad Prism 8.0 software (GraphPad Prism Software, CA, USA). Independent samples were analyzed with a two-tailed Student's t-test, while a paired t-test was used for paired samples. For comparisons involving three or more groups, we performed one-way ANOVA.

## Results

### *Mettl1^flox/flox^Prrx1^cre^* mice show skeletal deformities

**Figure 1:**
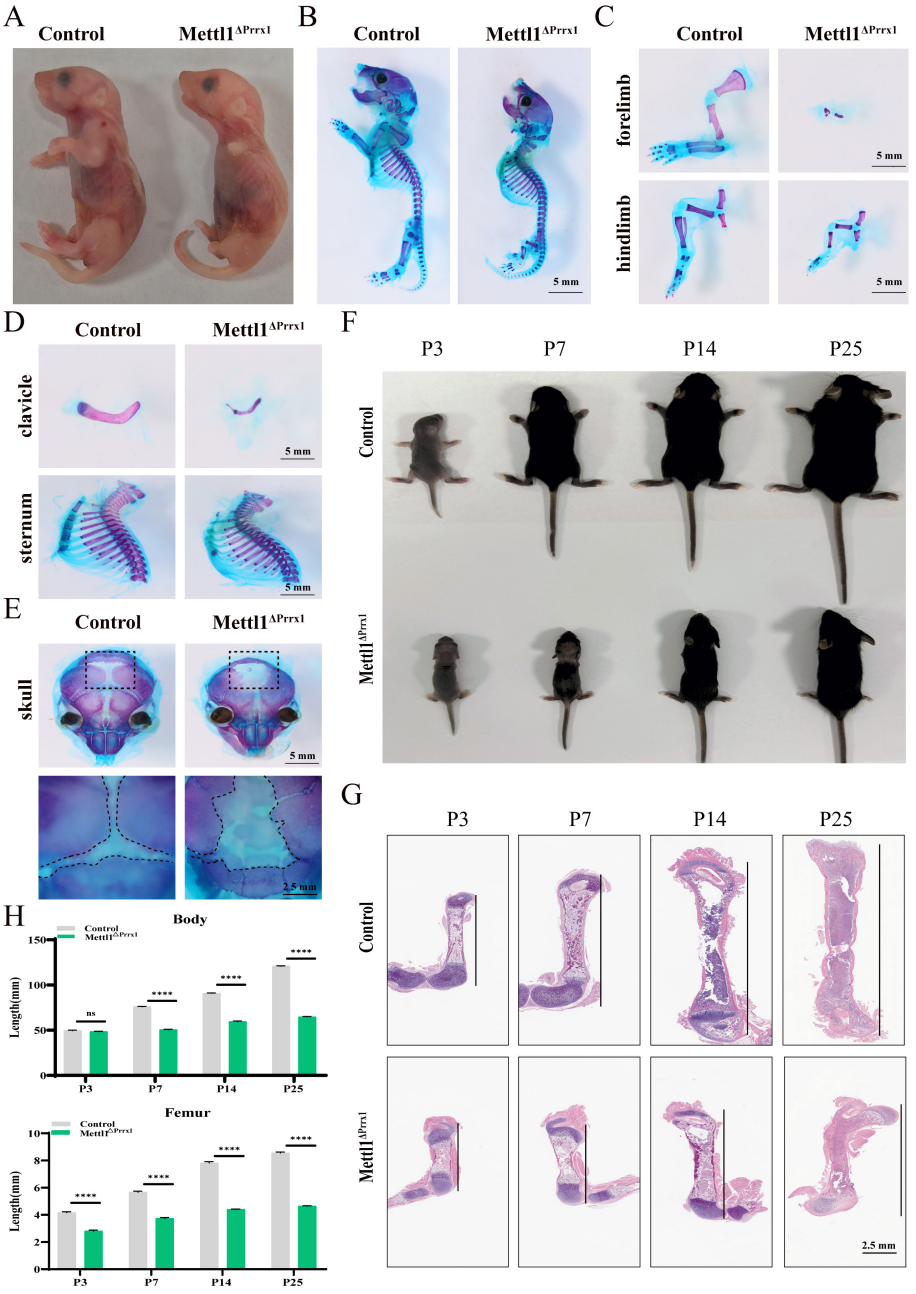
**Mettl1 deletion in *Prrx1*^+^ cell exhibits a skeletal dysplasia phenotype.** (A) Representative images of newborn *Mettl1^flox/flox^* mice (Control) and *Mettl1^flox/flox^ Prrx1^Cre^* (cko) mice at postnatal day 2 (P2). Scale bar = 5 mm. n = 3 per group. (B) Whole mount skeleton staining of newborn mice using Alcian blue-Alizarin red S. Scale bar = 5 mm. n = 3 per group. (C-E) Alcian blue-alizarin red S staining of limbs, clavicle, sternum and skull. Scale bar = 5mm or 2.5mm. (F) Representative images of Control and cko mice at P3, P7, P14 and P25. (G) H&E staining of femur tissues from Control and cko mice at P3, P7, P14 and P25. Scale bar = 2.5mm. (H) Quantification of the body length and femur length of indicated genotype mice (n = 3 per group). The statistical data are represented as the means ± SDs, ****P < 0.001, ns = no significant difference.

To investigate the role of Mettl1-mediated m^7^G methylation in skeletal development, we generated *Mettl1^flox/flox^Prrx1^cre^* mice (cko mice) to selectively eliminate Mettl1 in all mesenchymal cells (Supplementary Figure S1A). The effectiveness and specificity of the conditional knockout were confirmed through quantitative real-time PCR (qPCR) and western blot analyses (Supplementary Figure S1B and C). As illustrated in Figure 1A, cko mice exhibited absence of forelimbs and severely shortened hindlimbs at birth. Alcian blue and alizarin red staining of whole-mount skeletons revealed marked reductions in the lengths of the forelimbs, hindlimbs, and clavicle in cko mice, while no notable defects were observed in the sternum and spine (Figure 1B-D and Supplementary Figure S1D). Additionally, the skull was clearly underdeveloped in cko mice (Figure 1E). These abnormalities in cko mice indicated that Mettl1 is crucial for endochondral ossification which develops the limb and axial skeleton, as well as intramembranous ossification, primarily responsible for forming the skull and part of the clavicle. To further assess overall development, we monitored the body length of male mice from 3 to 25 days post-birth. We found that the growth rate of cko mice was markedly lower than that of control mice (Figure 1F). By postnatal day 25, cko mice showed stunted growth, with shorter body sizes and femur lengths, and a delayed closure of the fontanel, compared to control mice (Figure 1G-H; Supplementary Figure S1E). This reduced length gain was correlated with lower final weight and smaller individual organ (Supplementary Figure S1F). Collectively, these findings demonstrate that loss of Mettl1 in mesenchymal stem cell leads to critical abnormalities in skeletal development.

### Mettl1 deletion blocks chondrocyte and osteoblast differentiation

**Figure 2:**
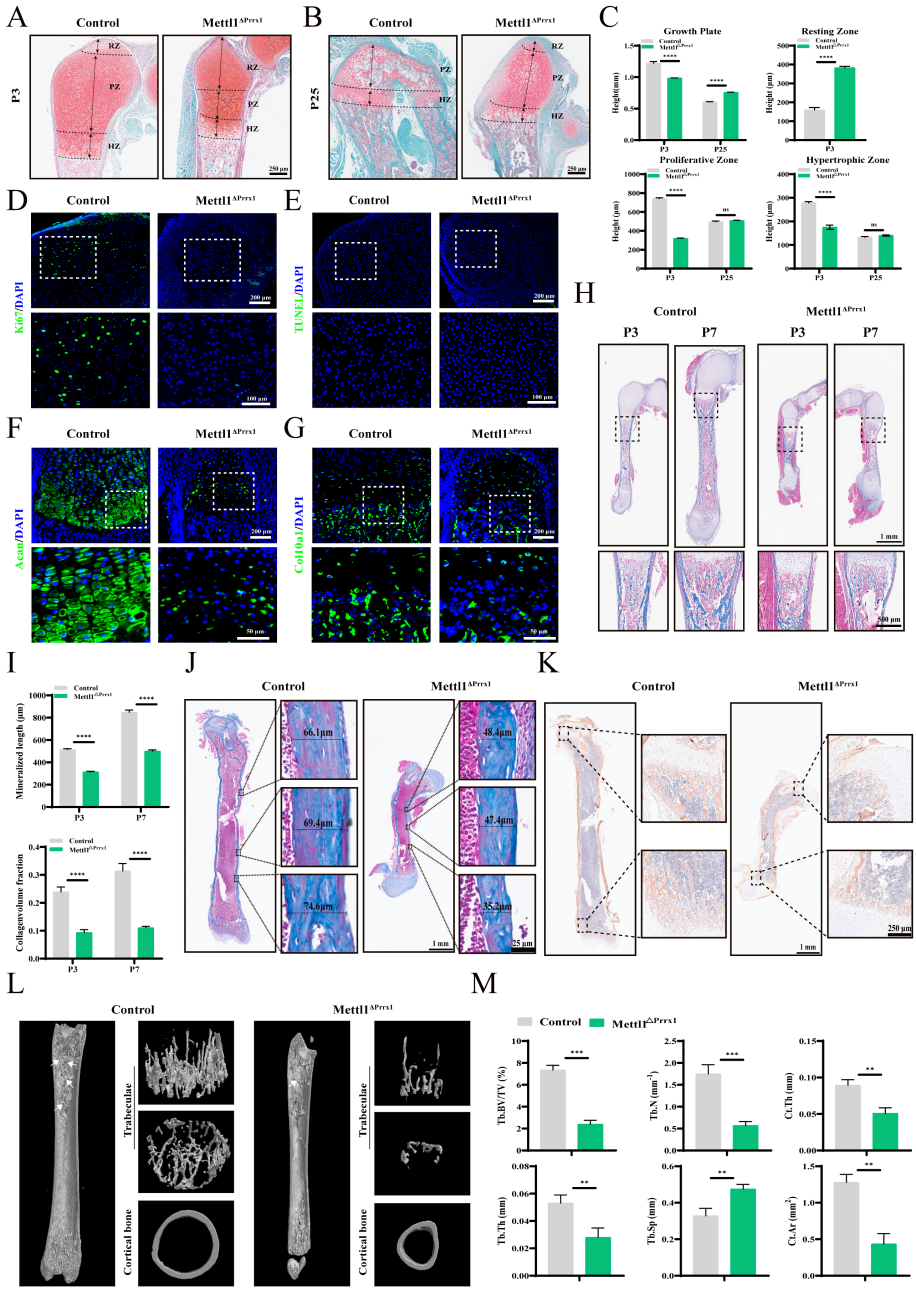
** Mettl1 deletion in Prrx1+ cell impairs chondrocyte and osteoblast differentiation.** (A and B) Safranin O/ Fast Green staining of femur tissues from Control and cko mice at P3 and P25. RZ, resting zone; PZ, proliferating zone; HZ, hypertrophic zone. Scale bar =250μm. (C) Quantification of the heights of growth plate, RZ, PZ, and HZ in femurs (n = 3 per group). (D) Representative immunostaining of ki67 expression in growth plates of Control and cko mice. Scale bar = 200μm or 100μm. (E) Cell death assessed by TUNEL assay in growth plates of Control and cko mice. Scale bar = 200μm or 100μm. (F and G) Representative immunostaining of Acan and Col10a1 expression in growth plates of Control and cko mice. Scale bar = 200μm or 50μm. (H) Masson staining of femur tissues from Control and cko mice at P3 and P7. Scale bar = 1mm or 500μm. (I) Quantification of mineralized length and collagen volume fraction of growth plates (n = 3 per group). (J) Representative Masson staining of femur tissues from Control and cko mice at P25. n = 3 per group. Scale bar = 1mm or 25μm. (K) Representative immunohistochemical staining of Col1a1 expression in femurs. n = 3 per group. Scale bar = 1mm or 250μm. (L) Representative micro-CT images of femurs from Control and cko mice at P25. From top to bottom, the image presents the plan view and top view of the three-dimensional reconstruction of the trabeculae, followed by the three-dimensional reconstruction of the cortical bone. The white arrows indicate the trabecular structures. (M) Quantification of bone morphometric analysis. Tb.BV/TV, trabecular bone volume per tissue volume; Tb.N, trabecular number; Tb.Th, trabecular thickness; Tb.Sp, trabecular bone separation; Ct.Th, cortical bone thickness; Ct.Ar, cortical bone area. Scale bar = 400μm. n = 3 per group. The statistical data are represented as the means ± SDs, **P < 0.01, ***P < 0.005, ****P < 0.001, ns = no significant difference.

We next conducted histological analyses of hindlimbs to assess the cellular abnormalities in the bones of cko mice. At P3, the growth plate of cko mice was shorter and exhibited a reduced number of chondrocytes compared to control mice. In the control group, there were significantly more chondrocytes located in the proliferation and hypertrophic zones, while in the cko group, more cells were found in the resting zone. At P25, the chondrocytes in the cko group began to exhibit proliferative activity, indicating that chondrocyte proliferation was limited in cko mice, which ultimately hindered the hypertrophic differentiation of cartilage (Figure 2A-C). Immunofluorescence analysis showed nearly undetectable levels of Ki67 in cko mice (Figure 2D; Supplementary Figure S1G). Unexpectedly, TUNEL staining revealed that Mettl1 knockout did not induce cell death (Figure 2E; Supplementary Figure S1H). Furthermore, hypertrophic differentiation markers like Acan and Col10a1, were markedly downregulated in cko mice (Figure 2F and G; Supplementary Figure S1I and J), implying that Mettl1 knockout inhibited chondrocyte hypertrophic differentiation. These findings indicate that Mettl1 knockout disrupts the chondrocyte differentiation in endochondral ossification process.

During the growth of long bones, hypertrophic chondrocytes undergo apoptosis, and hypertrophic cartilage is resorbed, allowing osteoprogenitor cells to infiltrate and differentiate into osteoblasts 21. This process leads to the formation of trabecular bone and the deposition of cortical bone around the cartilage matrix. As shown in Figure 2H, Masson staining revealed a notable decrease in the number of trabecular bones in cko mice. From P3 to P7, the expansion rate of the primary ossification center in cko mice was noticeably slower than in control mice (Figure 2I). Additionally, measurements of cortical bone thickness in the femurs of mice at P25 showed that cko mice exhibited notably thinner cortical bone (Figure 2J; Supplementary Figure S1K). These observations imply that Mettl1 deficiency impaired osteogenic differentiation and mineralization functions. Immunohistochemical analysis of Col1a1 revealed that the cells in the growth plates at both ends of the epiphysis in cko mice were functionally inactive, which hindered longitudinal bone growth (Figure 2K). Micro-CT scans provided strong evidence that the lack of Mettl1 severely compromised bone formation in these mice (Figure 2L and M). These results suggest that Mettl1 knockout adversely affected the differentiation of osteoblasts and their mineralization abilities. This implies that loss of Mettl1 also hinders osteoblast differentiation in intramembranous ossification process.

### Single-cell transcriptomic landscape reveals anomalies in lineage commitment within mesenchymal stem cell populations

**Figure 3:**
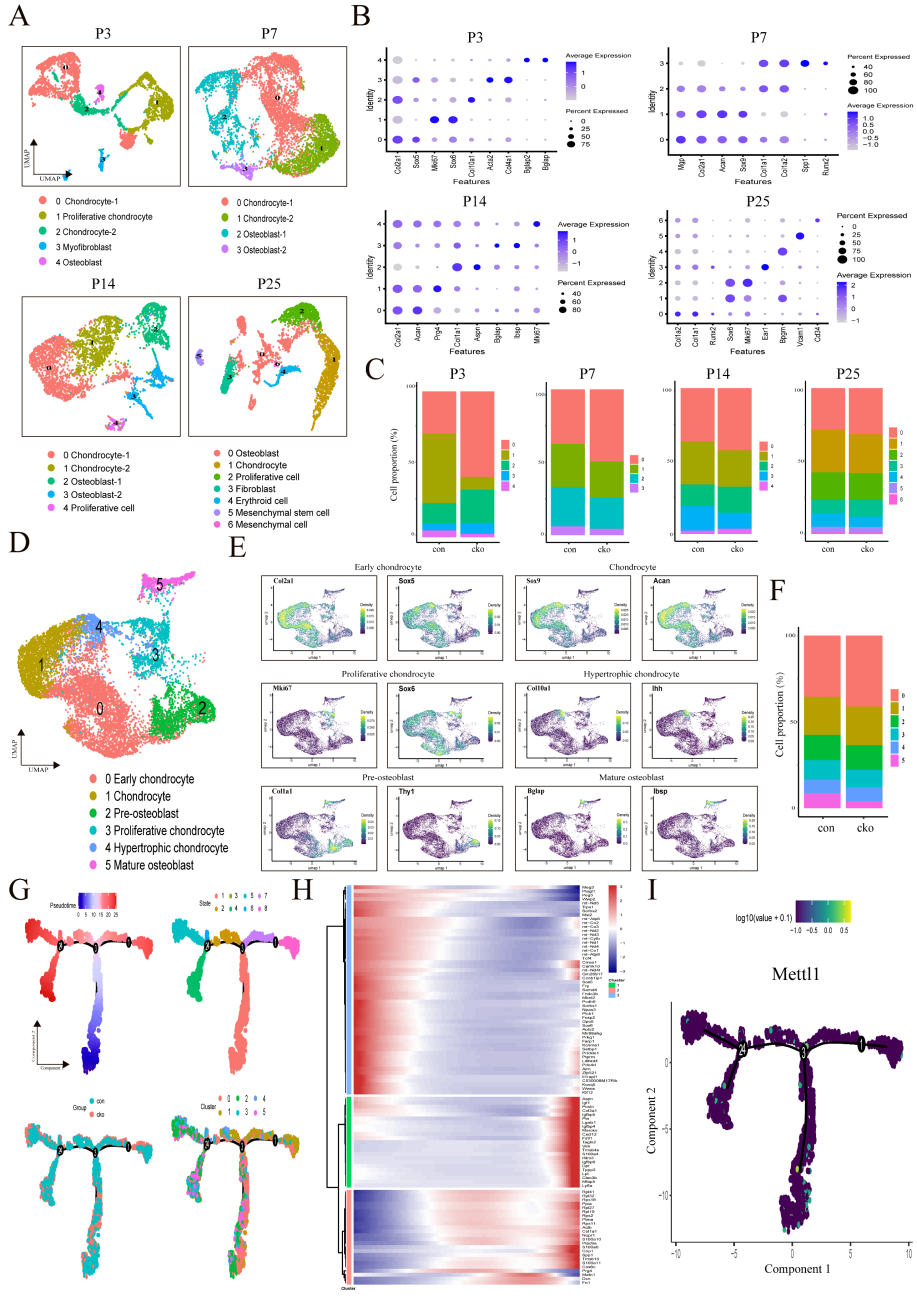
** Single-cell RNA sequencing analysis identifies alterations in mesenchymal lineages.** (A) UMAP projection of integrated scRNA-seq data, labeled by corresponding cell type, containing 3,622 (P3), 5,902 (P7), 3,632 (P14) and 4,608 (P25) cells. (B) Dot plot showing typical gene expression profiles of the identified cell clusters from Figure 3A. (C) Proportion of cell populations within each genotype. (D) UMAP projection of all mesenchymal lineage cluster, indicating four chondrocyte states and two osteoblast states. (E) UMAP representation of typical marker gene expression in each cluster from Figure 3D. (F) Percentage of cells represented by each cluster from Figure 3D. (G) Pseudotime trajectory of chondrocytes and osteoblasts with distinct color coding for pseudotime, state, group and cluster. (H) Heatmap depicting distinct gene expression signatures of three identified gene modules along mesenchymal lineages trajectory. (I) Expression patterns of Mettl1 along the inferred cell trajectory.

To compare the heterogeneity of mesenchymal lineage cells over time between control mice and cko mice, we collected eight fresh femur samples at four distinct time points. These tissues were dissociated into single cells, followed by high-throughput single-cell RNA sequencing (scRNA-seq) using the 10x Genomics platform. After rigorous quality control and batch removal, we successfully obtained 55,387 single cells. Utilizing the expression of cluster-specific markers 22-26, we categorized these cells into various types (Supplementary Figure S2A-D). Our analysis specifically focused on osteoblast and chondrocyte lineage cells, which were further classified based on gene expression patterns, including Col1a1, Bglap, Ibsp, Col2a1, Acan, and Sox9 (Figure 3A and B). Deletion of Mettl1 resulted in significant alterations in cell proportions (Figure 3C). Notably, at P3, the percentage of proliferative chondrocytes in cko mice was drastically reduced, corroborating our histological findings. As development progressed, this initial imbalance in chondrocyte proportions gradually diminished. Subsequently, we aggregated all mesenchymal lineage cells from the four time points and based on the canonical markers, categorized them into six distinct cell types: early chondrocyte, chondrocyte, proliferative chondrocyte, hypertrophic chondrocyte, pre-osteoblast, and mature osteoblast (Figure 3D and E). In both pre-osteoblast and mature osteoblast populations, the proportion of cells in the cko group was significantly lower than in the control group (Figure 3F), consistent with previous results.

To further investigate potential developmental differences, we conducted an analysis of cell differentiation trajectories in all mesenchymal cells using Monocle. The trajectories were categorized into four nodes and eight distinct states, with node 3 identified as a key transition point in the differentiation process (Figure 3G). Both control and cko groups demonstrated significant overlap in their differentiation trajectories. Throughout different time points, cells from both groups predominantly occupied similar nodes, indicating that the loss of Mettl1 did not alter the differentiation direction of mesenchymal cells (Supplementary Figure S3A). Additionally, we observed that early chondrocytes were mainly found at the start of the differentiation trajectory, with chondrocyte differentiation primarily following trajectory branch 1, while osteoblast differentiation occurred mainly along trajectory branch 4 (Supplementary Figure S3B). We also noted overlaps among proliferative chondrocytes, hypertrophic chondrocytes, and osteoblasts throughout the trajectory. This supported the notion that chondrocytes can transdifferentiate into osteoblasts after undergoing proliferation and hypertrophic differentiation during bone development 27. Importantly, cko mice displayed a significantly slower rate of differentiation compared to control mice (Supplementary Figure S3C). At P3, fewer cells in the cko group were found in the initial differentiation state (state 1), while at P25, a larger proportion of cells in the control group reached the final differentiation state (state 4). In contrast, the cko group retained more cells in the intermediate differentiation state (state 5). These observations suggested that Mettl1 knockout resulted in reduced speed of cell state transitions in mesenchymal lineage cells. To explore gene dynamics associated with differentiation trajectories, we identified pseudotime-dependent genes and categorized them into three modules based on their expression patterns (Figure 3H). Genes in module 1 and 2 demonstrated an increasing expression trend along the differentiation trajectory, while those in module 3 exhibited a decreasing trend. Upon comparing gene expression along the trajectory, we observed that downregulation of the chondrocyte marker genes expression (like Sox5 and Sox6) was accompanied by the upregulation of the osteoblast marker genes expression (like Col1a1). Additionally, we assessed the expression distribution of Mettl1 throughout the differentiation trajectory and found that Mettl1 was highly expressed at the early stages of differentiation (Figure 3I). This result indicates that Mettl1 may play a crucial role in regulating the activation and fate determination of mesenchymal lineage cells during bone development.

### Loss of Mettl1 disrupts m^7^G modifications of Fgfr2

**Figure 4:**
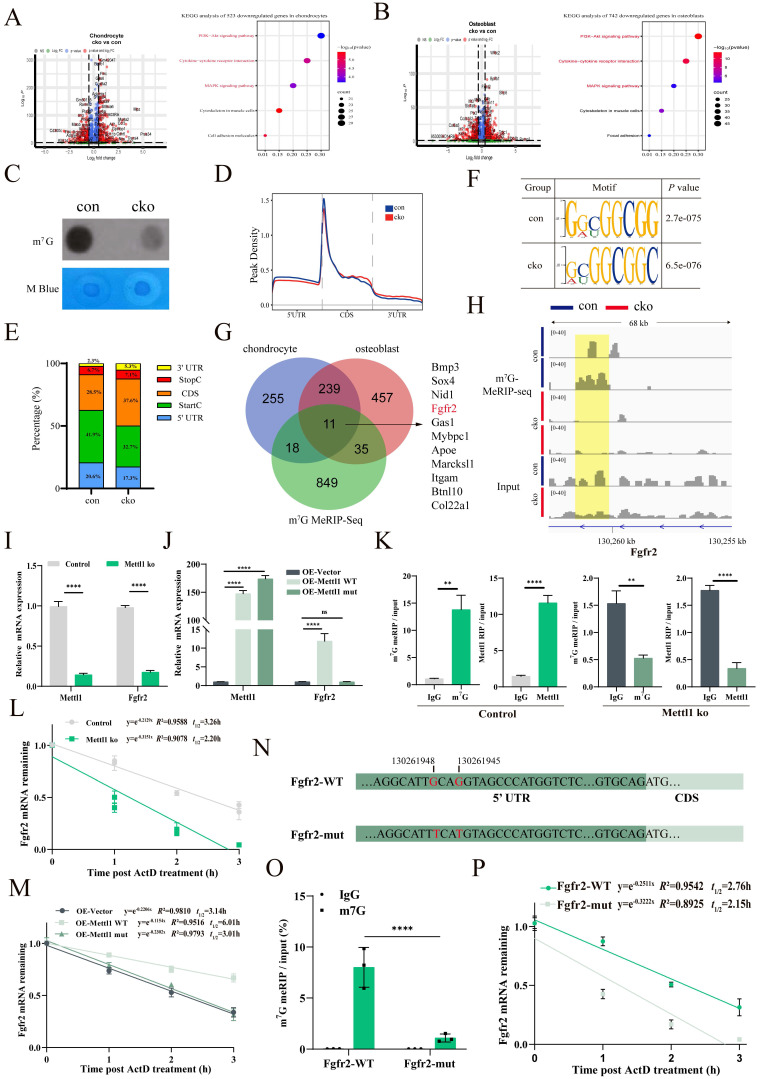
** Mettl1 deficiency disrupts m^7^G modifications of Fgfr2.** (A) Volcano plot showing differential gene expression in chondrocytes and dot plot of enrichment analysis for downregulated genes in chondrocytes. (B) Volcano plot showing differential gene expression in osteoblasts and dot plot of enrichment analysis for downregulated genes in osteoblasts. (C) Dot blot analysis of m^7^G modification levels in femurs of mice at P3, with methylene blue (M Blue) staining as control. (D) Density distribution of m^7^G modifications across mRNA segments in the m^7^G MeRIP-seq data. (E) A histogram depicting the regional distribution of m^7^G sites identified by MeRIP-seq. (F) Top motifs and their p-values identified from m^7^G MeRIP-seq data in femur tissues of Control and cko mice. (G) A Venn diagram showing the overlap between decreased m^7^G peaks and reduced RNA expression analyzed by m^7^G MeRIP-seq and sc-RNA seq. (H) Integrative Genomics Viewer (IGV) analysis of the m^7^G peaks of Fgfr2. The orange rectangular shading represents the distribution of the m7G peak of Fgfr2 across the different samples. (I) The expression levels of Mettl1 and Fgfr2 mRNA were measured in primary mice MSCs of control group and Mettl1 ko group (n=3). (J) The expression levels of Mettl1 and Fgfr2 mRNA were measured in primary mice MSCs of overexpression (OE)-vector group, oe-Mettl1 WT group and oe-Mettl1 mut group (n=3). The Mettl1 mutant plasmid lacks methyltransferase activity (AA160-163: LFPD to AFPA). (K) Validation of the m7G enrichment on Fgfr2 mRNA by RIP-qPCR in primary mice MSCs of control group and Mettl1 ko group (n=3). (L and M) RNA stability assay to detect the mRNA half-lives of Fgfr2 by qPCR (n=3). (N) A graphical illustration of the construction of the plasmid carrying Fgfr2 mutation sites (G-to-T mutation). (O) Fgfr2 wild-type (Fgfr2-WT, 5′UTR + CDS) or Fgfr2-mutant (Fgfr2-Mut, 5′UTR-mutant + CDS) plasmids were co-transfected with oe-Mettl1 WT into primary mice MSCs, respectively. The effect of Mettl1 on m7G modification of exogenous Fgfr2 transcript was assessed by RIP-qPCR. (P) The decay rates of exogenous Fgfr2 transcript analyzed by qRT-PCR in primary mice MSCs treated with actinomycin D (5 μg/ml) for 0, 1, 2, and 3 h after transfection with oe-Mettl1 WT for 48 h (n = 3). The statistical data are represented as the means ± SDs, **P < 0.01, ****P < 0.001, ns = no significant difference.

To explore the transcriptional landscape of cko mice, we specifically focused on clusters of chondrocytes (early chondrocyte, chondrocyte, proliferative chondrocyte and hypertrophic chondrocyte) and osteoblasts (pre-osteoblast, and mature osteoblast). In chondrocytes, differential expression gene (DEG) analysis revealed a total of 1113 genes, comprising 590 upregulated and 523 downregulated genes (Figure 4A). Kyoto Encyclopedia of Genes and Genomes (KEGG) analysis of downregulated genes demonstrated that PI3K-AKT, cytokine-cytokine receptor interaction and MAPK signaling pathways were the top 3 enriched pathways. In osteoblasts, we detected a total of 1464 DEGs, which included 722 upregulated and 742 downregulated genes (Figure 4B). Similar to the results of chondrocytes, KEGG analysis of downregulate genes in osteoblast showed the same top enriched pathways. As canonical upstream regulators of the PI3K-AKT and MAPK signaling cascades, receptor tyrosine kinases (RTKs) have been mechanistically implicated in MSCs differentiation and the processes of skeletal patterning 28, 29. To elucidate the mechanism by which Mettl1 regulate MSCs differentiation through RTKs, we performed m^7^G MeRIP-seq on the femoral tissue. Total m^7^G methylation levels in cko mice femoral tissue were markedly lower compared to control mice (Figure 4C). Since mRNA internal m^7^G is reported to be enriched at 5′untranslated region (5′UTR) near the translation initiation site 17, our experiments showed that Mettl1 deletion in Prrx1^+^ cells led to a substantial reduction in m^7^G peaks at 5′UTR and Start C (Figure 4D and E). Interestingly, both control and cko mice exhibited a similar enrichment of mRNA internal m^7^G, with the predominant motif being “GGCGG”(Figure 4F). Given that mRNA internal m7G is known to be enriched in a GA-rich context, the second most common motif was identified as “GGARGA”. By intersecting the m^7^G MeRIP-seq results with scRNA-seq data from bone tissues, we identified 11 genes that displayed decreased m^7^G peaks and reduced RNA expression following Mettl1 deletion (Figure 4G). Notably, we found that fibroblast growth factor receptor 2 (Fgfr2), the common upstream RTKs of PI3K-AKT and MAPK pathways, was listed in these 11 genes. Integrative Genomics Viewer analysis further revealed markedly decreased m^7^G levels of Fgfr2 in cko group (Figure 4H). Thus, we speculated that Mettl1 regulates MSCs differentiation by modulating m^7^G modifications of Fgfr2.

We then isolated primary mice MSCs from femoral tissues of *Mettl1^flox/flox^* mice. Mettl1 knockout and overexpression cell models were constructed by using cre-expression adenoviruses and Mettl1-overexpression adenoviruses, respectively. qPCR results validated that mRNA levels of Fgfr2 decreased following Mettl1 knockout (Figure 4I). To determine whether these changes were dependent on m^7^G modifications catalyzed by Mettl1, we generated a Mettl1-mutant plasmid lacking methyltransferase activity (AA160-163: LFPD to AFPA) 18. Overexpression of wild-type Mettl1 increased mRNA levels of Fgfr2, while overexpression of mutant Mettl1 had no effect (Figure 4J). To examine the role of Mettl1 in mediating m^7^G modifications of Fgfr2, RNA immunoprecipitation (RIP) assays using either m7G or Mettl1 antibody after qPCR confirmed that Mettl1 directly interacted with the m^7^G modification sites of Fgfr2 (Figure 4K). Furthermore, mRNA stability assays demonstrated that the half-life of Fgfr2 mRNA was markedly reduced in Mettl1 knockout cells (Figure 4L). In Mettl1 overexpression cells, wild-type Mettl1 significantly extended the half-life of Fgfr2 mRNA, whereas the mutant form did not (Figure 4M). To further confirm that Fgfr2 was a direct target of Mettl1, we mutated the m7G sites of Fgfr2 and constructed plasmids containing wild-type Fgfr2 5′UTR regions (Fgfr2-WT) or mutant Fgfr2 5′UTR (Fgfr2-mut) (Figure 4N). As anticipated, m^7^G RIP-qPCR results showed that Fgfr2-WT, but not Fgfr2-mut, was able to be immunoprecipitated by anti-m^7^G (Figure 4O). Additionally, the mRNA stability of Fgfr2-mut was markedly lower than that of Fgfr2-WT (Figure 4P). Collectively, these findings indicate that Mettl1 stabilizes Fgfr2 mRNA through m^7^G modifications.

### Mettl1 deletion inactivates PI3K-AKT and MAPK pathways by reducing Fgfr2

**Figure 5:**
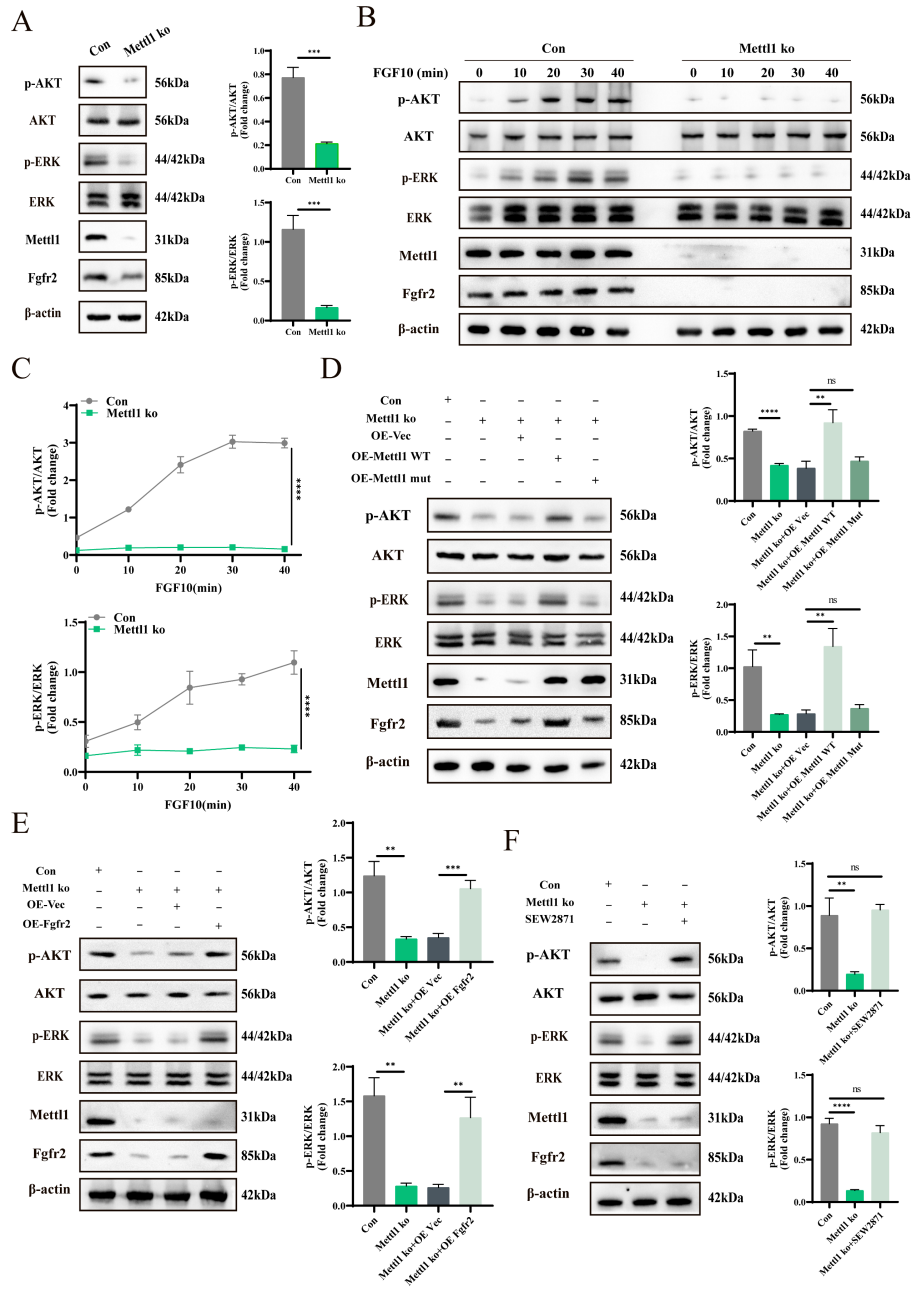
** Mettl1 deletion inactivates PI3K-AKT and MAPK pathways by reducing Fgfr2.** (A) Western blot analysis of AKT and ERK activation in primary mice MSCs with/without Mettl1 ko (control group and Mettl1 ko group). The histogram showed the fold change in AKT and ERK activation (n = 3). (B) Western blot analysis of AKT and ERK activation in control group and Mettl1 ko group with or without FGF10 (50 ng/ml) stimulation. (C) The curve chart shows the fold change in AKT and ERK activation (n = 3). (D and E) The protein levels of AKT and ERK in primary mice MSCs transfected with the indicated constructs. (F) Western blot analysis of AKT and ERK activation in control group and Mettl1 ko group with or without SEW2871 (0.5 μM/ml). The statistical data are represented as the means ± SDs, ** P < 0.01, *** P < 0.005, **** P < 0.001, ns = no significant difference.

To investigate the role of Mettl1 in modulating the PI3K-AKT and MAPK signaling pathways via Fgfr2, we conducted rescue experiments. First, we observed a significant downregulation of p-AKT and p-ERK expression in the Mettl1 ko group (Figure 5A). Consistently, Mettl1 depletion resulted in delayed activation of AKT and ERK signaling following stimulation with the FGFR2 ligand FGF10 in primary mice MSCs (Figure 5B and C).

Moreover, only the reintroduction of Mettl1 WT was able to restore AKT and ERK signaling, whereas Mettl1 mutant group failed to produce this effect (Figure 5D). This further supports the notion that Mettl1 regulates the activation of the PI3K-AKT and MAPK signaling pathways through the m^7^G modification of Fgfr2. Additionally, we found that overexpressing Fgfr2 could partially rescue the inhibitory effects of Mettl1 deletion (Figure 5E). To further investigate whether activation of the Fgfr2 signaling pathway could mitigate the Mettl1 knockout phenotype, we treated the cells with SEW2871, an agonist known to activate AKT and ERK. SEW2871 treatment effectively reactivated the AKT and ERK pathways in the Mettl1 ko group (Figure 5F). Collectively, these findings suggest that Mettl1 modulates the PI3K-AKT and MAPK signaling pathways through the regulation of Fgfr2.

### Mettl1 deficiency decreases Sp1 phosphorylation and expression through Fgfr2 signaling inactivation

**Figure 6:**
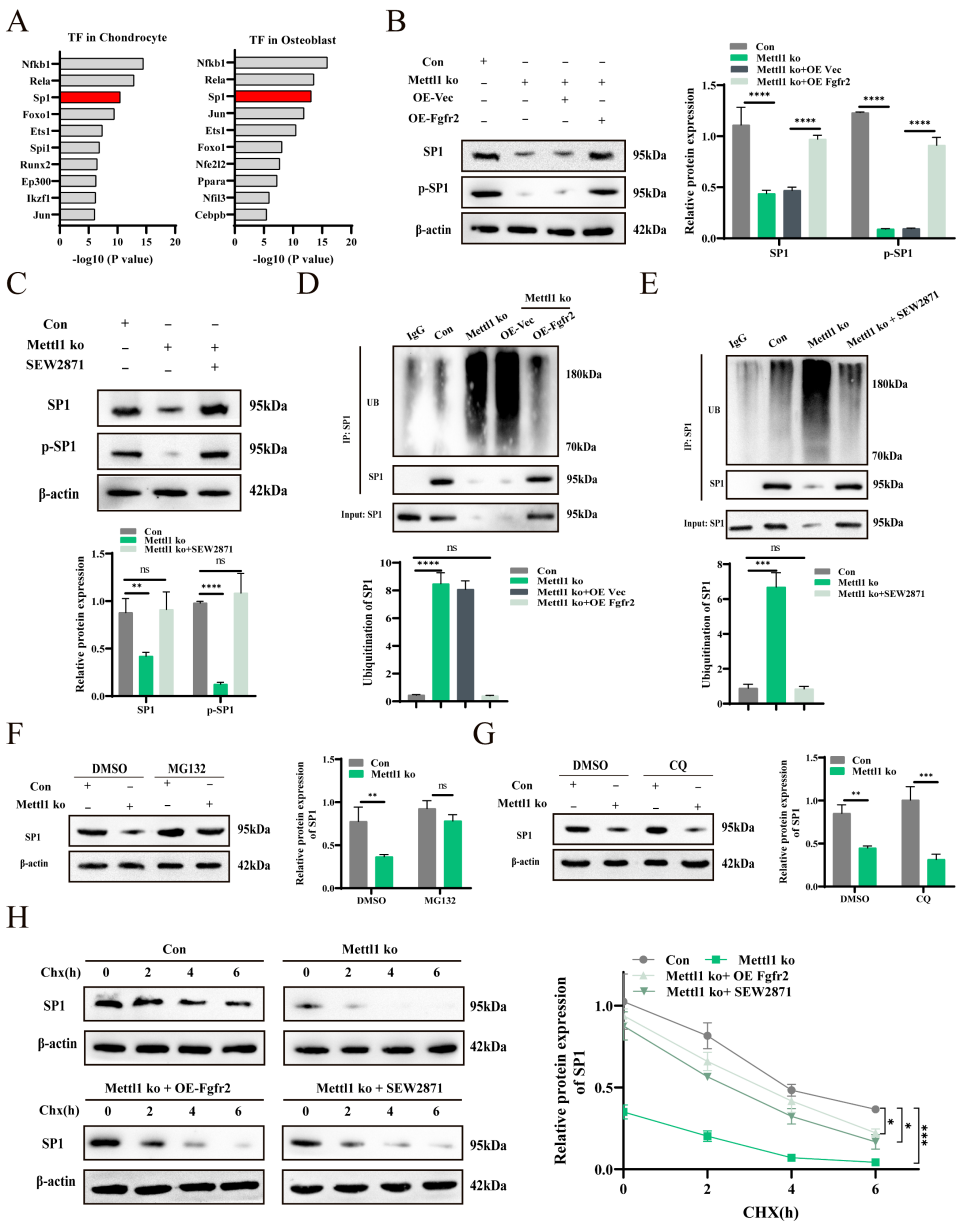
** Mettl1 deficiency decreases Sp1 phosphorylation and expression through Fgfr2 signaling inactivation.** (A) Top transcription factor-regulated differentially expressed genes in chondrocytes and osteoblasts. (B) The protein levels of Sp1 and p-Sp1 in primary mice MSCs transfected with the indicated constructs. (C) The protein levels of Sp1 and p-Sp1 in control group and Mettl1 ko group with or without SEW2871 (0.5 μM/ml). (D) Immunoprecipitation (IP) analysis of the ubiquitination of Sp1 in control group and Mettl1 ko group transfected with the indicated constructs. (E) IP analysis of the ubiquitination of Sp1 in control group and Mettl1 ko group with or without SEW2871 (0.5 μM/ml). (F and G) Western blot analysis of Sp1 stability in control group and Mettl1 ko group upon treatment with the protease inhibitor MG132 or the lysosome inhibitor chloroquine (CQ). (H) Protein level of Sp1 in control group and Mettl1 ko group upon treatment with the protein translation inhibitor CHX. The line chart shows the expression and degradation rate of Sp1. n = 3 in each group. The statistical data are represented as the means ± SDs, *P < 0.05, ** P < 0.01, *** P < 0.005, **** P < 0.001, ns = no significant difference.

To explore the relationship between Fgfr2 signaling inactivation and gene transcription regulation under Mettl1 deficiency, we performed transcription factor enrichment analysis on downregulated gene sets from chondrocytes and osteoblasts. The analysis identified Sp1 as a key target of many differentially expressed genes (Figure 6A). Previous studies have shown that the phosphorylation level and expression of Sp1 are important downstream responses in the PI3K-AKT and MAPK signaling cascades 30, 31. Western blot analysis revealed a reduction in both Sp1 and p-Sp1 levels following Mettl1 knockout (Figure 6B and C). However, overexpression of Fgfr2 or SEW2871 treatment reversed this effect, suggesting that Mettl1 knockout may reduce Sp1 phosphorylation and regulate gene transcription via Fgfr2 signaling inactivation. Moreover, our previous work demonstrated that decreased Sp1 phosphorylation enhances its ubiquitination and subsequent degradation 32. Immunoprecipitation experiments showed increased Sp1 ubiquitination following Mettl1 knockout, while Fgfr2 overexpression or SEW2871 treatment inhibited Sp1 ubiquitination and degradation (Figure 6D and E). To further elucidate the mechanism by which Mettl1 regulates Sp1 stability, we used the proteasome inhibitor MG132 and the lysosome inhibitor CQ (Figure 6F and G). MG132 significantly increased Sp1 protein levels in the Mettl1 knockout group, while CQ had minimal effect, indicating that Mettl1 knockout promotes Sp1 degradation via the ubiquitin-proteasome pathway. Additionally, the protein translation inhibitor CHX showed that Sp1 stability was significantly reduced upon Mettl1 knockout (Figure 6H). Collectively, these findings suggest that Mettl1 knockout reduces Sp1 phosphorylation, enhances Sp1 ubiquitination and degradation, and ultimately affects the transcription of downstream target genes.

### Mettl1 deficiency suppresses Sp1-mediated Col1a1 and Col2a1 transcription

**Figure 7:**
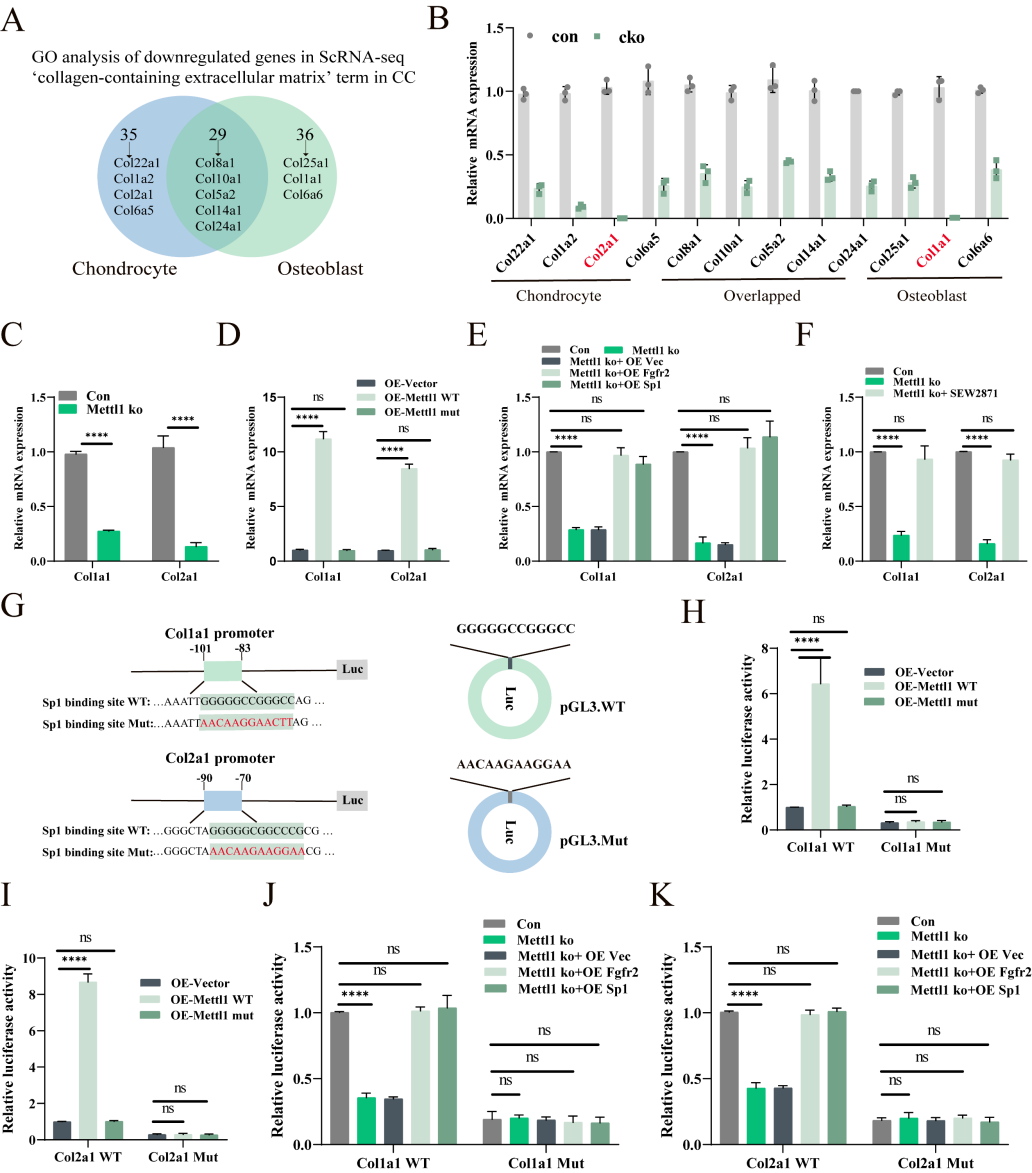
** Mettl1 deficiency disrupts Col1a1 and Col2a1 expression by decreasing Sp1 transcription.** (A) A Venn diagram showing the overlap between chondrocytes and osteoblasts in the 'collagen-containing extracellular matrix' term in the cellular component part of GO analysis. (B) The mRNA expression levels of collagen-related genes measured in femur tissues of Control and Mettl1^△Prrx1^ mice at P3 by qPCR (n=3 each group). (C) The mRNA expression levels of Col1a1 and Col2a1 measured in primary mice MSCs of control group and Mettl1 ko group (n=3). (D) The mRNA expression levels of Col1a1 and Col2a1 measured in primary mice MSCs of overexpression (OE)-vector group, oe-Mettl1 WT group and oe-Mettl1 mut group. (E and F) The mRNA expression levels of Col1a1 and Col2a1 measured in primary mice MSCs transfected with the indicated constructs. (G) Schematic diagram of Sp1 binding to the Col1a1 and Col2a1 gene promoters and the putative binding sites in the promoter regions. (H-K) Either the WT or Mut Col1a1/Col2a1 promoter reporter plasmids were transfected into cells and the dual-luciferase assay was employed to assess the activity of the promoter after 48h. n = 3 per group. The statistical data are represented as the means ± SDs, *P < 0.05, **P < 0.01, ***P < 0.005, ****P < 0.001, ns = no significant difference.

To investigate the Sp1-mediated transcriptional changes triggered by Mettl1 deficiency, we performed GO enrichment analysis on the differentially expressed genes in chondrocytes and osteoblasts. Lollipop chart results indicated that most of the enriched genes were collagen-related (Supplementary Figure S3D and E). We then compared the enrichment results between the two cell types and identified 11 collagen-associated genes (Figure 7A). qPCR analysis revealed that all 11 collagen-related genes were downregulated in the bone tissues of cko mice, with the most pronounced downregulation observed in chondrocyte-specific Col2a1 and osteoblast-specific Col1a1 (Figure 7B). Previous studies have shown that Sp1 can bind to the promoters of Col1a1 and Col2a1 to regulate their expression 33, 34. Based on this, we hypothesize that Mettl1 regulates the expression of Col1a1 and Col2a1 through Sp1 to modulate osteogenic and chondrogenic differentiation of mesenchymal stem cells. In line with this hypothesis, the expression levels of Col1a1 and Col2a1 were downregulated in Mettl1 knockout cells and upregulated in Mettl1-WT overexpressing cells, while no upregulation was observed in the Mettl1 mutant overexpression group (Figure 7C and D).

Supplementation experiments demonstrated that overexpression of Fgfr2 and Sp1 could reverse the inhibitory effect of Mettl1 deficiency on Col1a1 and Col2a1 expression (Figure 7E). Treatment with SEW2871 produced similar results (Figure 7F), suggesting that Mettl1 deficiency suppresses Col1a1 and Col2a1 expression via the Fgfr2-Sp1 axis. We constructed luciferase reporter plasmids containing the Col1a1 and Col2a1 promoters, including wild type and mutant versions (Figure 7G). Dual-luciferase reporter assays revealed that overexpression of Mettl1 WT significantly increased the transcription of Col1a1 and Col2a1, whereas Mettl1 mutant did not (Figure 7H). Mettl1 knockout also significantly reduced transcriptional activity of Col1a1 and Col2a1, but supplementation with Fgfr2 and Sp1 reversed this effect (Figure 7I-K). The mutant group abolished Col1a1 and Col2a1 promoter induction. Collectively, these findings suggest that Mettl1 regulates the transcription of Col1a1 and Col2a1 through the Fgfr2-Sp1 pathway.

### Fgfr2 signaling reactivation ameliorates damage caused by Mettl1 deletion

**Figure 8:**
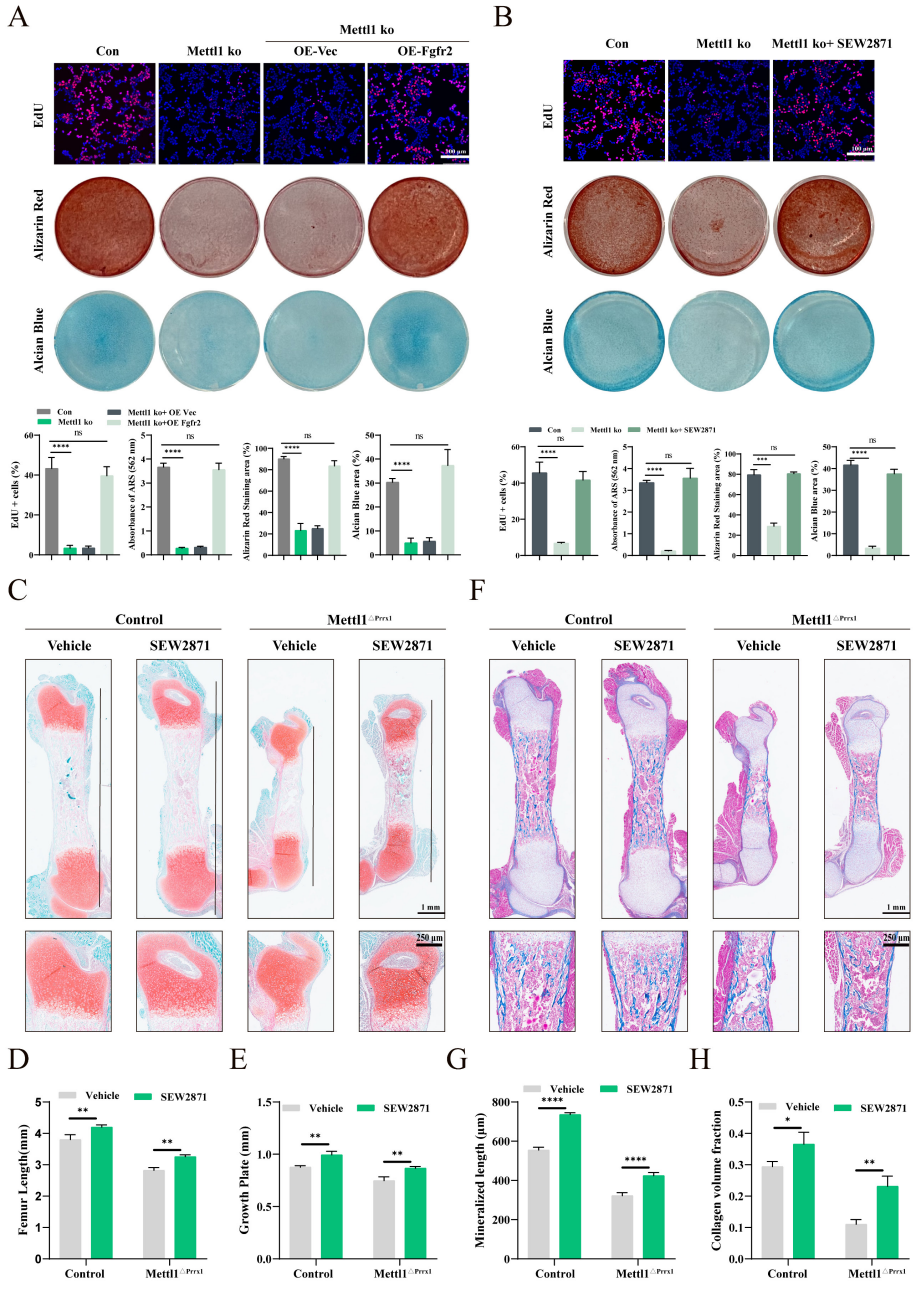
** Fgfr2 signaling reactivation ameliorates damage caused by Mettl1 deletion.** (A) Edu, alizarin red and alcian blue staining of primary mice MSCs transfected with the indicated constructs. Scale bar =100 μm. (B) Edu, alizarin red and alcian blue staining of control group and Mettl1 ko group with or without SEW2871 treatment. Scale bar =100 μm. (C) Safranin O/ Fast Green staining of femur tissues from Control and Mettl1^△Prrx1^ mice at P3 with or without SEW2871 treatment. Scale bar =1mm or 250μm. (D and E) Quantification of femur length and growth plate in indicated genotype mice. (F) Masson staining of femur tissues from Control and Mettl1^△Prrx1^ mice at P3 with or without SEW2871 treatment. Scale bar =1mm or 250μm. (G and H) Quantification of mineralized length and collagen volume fraction of growth plates. n = 3 per group. The statistical data are represented as the means ± SDs, *P < 0.05, **P < 0.01, ***P < 0.005, ****P < 0.001, ns = no significant difference.

We further explored whether reactivating Fgfr2 signaling could mitigate the defects caused by Mettl1 deletion. The Edu assay results indicated that overexpression of Fgfr2 or treatment with SEW2871 effectively rescued the proliferative defects in Mettl1 knockout cells (Figure 8A and B). Alizarin Red and Alcian Blue staining results showed that Fgfr2 signaling reactivation restored both osteogenic and chondrogenic differentiation of MSCs. To evaluate the effects of Fgfr2 signaling reactivation *in vivo*, pregnant mice were supplemented with SEW2871 in drinking water. Histological analysis revealed that SEW2871 treatment resulted in longer femora development and increased growth plate height in both control and cko mice (Figure 8C-E). Masson staining of femoral tissue further demonstrated that SEW2871 promoted bone mineralization and enhanced trabecular bone formation (Figure 8F-H). Collectively, these findings suggest that reactivation of Fgfr2 signaling can rescue the osteogenic and chondrogenic differentiation capacities of MSCs in Mettl1 cko mice.

## Discussion

Bone development is a multifaceted and dynamic process that encompasses growth, repair, and remodeling, mediated by various intracellular signaling pathways and growth factors 35, 36. Prrx1^+^ mesenchymal cells are notable for their self-renewal and multilineage differentiation potential, serving as a primary source for skeletal lineage cells 37, 38. These cells are capable of differentiating into chondrocytes, osteoblasts, and osteocytes, thereby playing a crucial role in maintaining bone homeostasis 39-41. Recent research has highlighted the importance of RNA modifications in regulating key genes within mesenchymal stem cells by influencing RNA stability, localization, turnover, and translation efficiency, which in turn impacts bone development and systemic equilibrium 42, 43. For instance, Yang et al. reported that NSUN4-mediated m^5^C and METTL3-mediated m^6^A modifications are essential for chondrocyte differentiation, as they collaboratively regulate the translation of Sox9 44. Furthermore, the knockout of Mettl3 in mesenchymal cells has been shown to decrease the translation efficiency of Pth1r, resulting in impaired osteogenic differentiation and bone formation 45. Our previous study indicated that the deletion of Alkbh5 in mesenchymal stem cells enhances bone mass in mice by modulating the mRNA stability of PRMT6 46. In the present study, we generated Mettl1 knockout mice to explore the role of m^7^G modification in mesenchymal linage cells. Our findings unequivocally demonstrated that Mettl1-mediated m^7^G modification is essential for osteogenic and chondrogenic differentiation of mesenchymal stem cells during endochondral ossification and bone ossification. At the single-cell level, Mettl1 knockout inhibited both the proliferation and hypertrophic differentiation of chondrocytes, as well as suppressed osteogenic differentiation. This inhibition of differentiation in these two cell types directly resulted in delayed cartilage and bone formation, ultimately undermining overall developmental integrity.

The m^7^G modification is primarily located in mRNA caps, internal mRNA, tRNA, rRNA and pri-miRNA 17, 47-49, playing essential roles in mRNA transcription, tRNA stability, rRNA maturation, and miRNA biogenesis. Extensive studies on the m^7^G modification of tRNA have demonstrated its considerable influence on processes such as stem cell differentiation 18, vascular development 50, aging 51, and tumor progression 52-54, largely by affecting translation efficiency. Studies have shown that m^7^G modification of internal mRNA is predominantly concentrated in the AG region of the 5′ UTR, which enhances mRNA translation 55. Recently, Liu et al. revealed that IGF2BP3 binds to internal m^7^G modifications in mRNA, facilitating its degradation 56. Yu et al. reported that Mettl1 drives cardiac hypertrophy by modulating the stability of SRSF9 mRNA 57. Zhang et al. showed that Mettl1 promotes the progression of castration-resistant prostate cancer (CRPC) by stabilizing CDK14 mRNA 58.

Fgfr2, expressed on mesenchymal cells during the mesenchymal condensation phase of bone formation, plays a critical role in skeletal development by initiating intracellular signaling cascades, including the PI3K/AKT, MAPK, JNK, and p38 pathways 59. In our study, we demonstrated Mettl1 deletion impaired the mRNA stability of Fgfr2 and subsequently deactivated Fgfr2 signaling.

Previous studies have shown that Sp1 phosphorylation not only modulates its transcriptional activity but also impacts its stability 60. Several kinases within the Fgfr2 signaling pathway are capable of directly phosphorylating Sp1 61. In the present study, we found that the inactivation of the Fgfr2 pathway results in reduced Sp1 phosphorylation and increased ubiquitination, ultimately promoting Sp1 degradation. Sp1 is known to activate the transcription of various cell-specific genes and plays a vital role in cell growth, differentiation, and apoptosis 62. Therefore, the downregulation of Sp1 transcriptional activity may be a key mechanism underlying the inhibition of mesenchymal cell differentiation in Mettl1 cko mice. Furthermore, our results revealed that Col1a1 and Col2a1 are significant genes regulated by Sp1. Col1a1 is mainly secreted by osteoblasts, while Col2a1 is secreted by chondrocytes. Due to the specificity of cellular secretion, Col1^+^ and Col2^+^ are widely recognized as markers for osteoprogenitor and chondroprogenitor cells 63, respectively. Current research on skeletal development defects has primarily focused on mutations or deletions within collagen genes 64. Our findings offer novel perspectives into skeletal diseases associated with the diminished expression of collagen genes.

Findings in this study open new possibilities for clinical applications in skeletal biology and regenerative medicine. Targeting Mettl1 or m^7^G modifications, for example, may provide a novel therapeutic strategy for skeletal disorders associated with impaired bone or cartilage formation, such as osteogenesis imperfecta or chondrodysplasia. Specifically, the ability to reactivate Fgfr2 signaling with small molecules like SEW2871 indicates that pharmacological modulation of this pathway could potentially correct bone and cartilage defects in patients with Mettl1 deficiency or related genetic mutations. This approach may be particularly valuable in pediatric patients with congenital skeletal deformities, where early intervention could prevent long-term complications such as stunted growth or skeletal fragility. Additionally, the discovery that m^7^G modifications stabilize Fgfr2 mRNA presents a potential diagnostic biomarker for skeletal diseases. Measuring m^7^G levels in patient-derived mesenchymal stem cells or circulating RNA could aid in identifying individuals at risk for skeletal abnormalities, allowing for early diagnosis and personalized treatment strategies. Moreover, gene therapy aimed at restoring Mettl1 function or enhancing m^7^G modifications in MSCs could provide a long-term solution for skeletal regeneration in patients with severe bone or cartilage defects. Finally, the involvement of Sp1 phosphorylation in regulating collagen expression (Col1a1 and Col2a1) suggests that targeting Sp1 stability or activity could offer another therapeutic approach. For instance, proteasome inhibitors that prevent Sp1 degradation may be repurposed to enhance collagen production in patients with skeletal disorders.

The Runt-related transcription factor (Runx) family plays a critical and indispensable role in the development, homeostasis, and regeneration of cartilage and bone 65. Among the Runx family members, Runx2 is widely recognized as the master regulator of osteoblast differentiation and chondrocyte maturation, essential for proper bone formation and skeletal integrity 66. Runx3, on the other hand, is involved in regulating aggrecan expression, a key component of cartilage, and plays an important role in the differentiation and maturation of chondrocytes 67. Given the pivotal roles of these transcription factors, we sought to investigate the effects of Mettl1 deletion on their expression. To this end, we analyzed the expression of both Runx2 and Runx3 in Mettl1 deletion contexts using Western blotting (Supplementary Figure S4A-B). The results revealed a significant reduction in Runx2 expression in bone tissue, while Runx3 expression was mildly downregulated. Similarly, in Mettl1 KO cells, both Runx2 and Runx3 levels were markedly reduced. In rescue experiments, overexpression of Fgfr2 or treatment with SEW2871 slightly increased Runx2 expression, but had no effect on Runx3 expression. Previous research suggests that Mettl1 may regulate Runx2 mRNA levels by modulating the m^7^G modification of Runx2 68. Additionally, the Fgf-MAPK signaling pathway has been reported to sustain Runx2 protein stability by enhancing its acetylation and preventing its ubiquitination 69. Based on these observations, we propose that the loss of Mettl1 alters Runx2 RNA expression, while the inactivation of Fgfr2 signaling leads to reduced stability of Runx2 protein. In our experiments, restoring Fgfr2 expression rescued differentiation defects but did not affect Runx3 levels. This suggests that the Mettl1-Fgfr2 axis regulates MSC differentiation independently of Runx3, and that the downregulation of Runx3 is not simply due to the suppression of Fgfr2, but may involve other, potentially direct post-transcriptional mechanisms related to m^7^G modification.

RNA modifications are reversible processes that are dynamically regulated by writers, readers, and erasers. Currently, the writer proteins involved in m^7^G modification are well characterized, and studies have identified QKI and IGF2BP3 as potential reader proteins that modulate these modifications 56, 70. However, specific eraser proteins for m^7^G modifications have not yet been reported. This research is limited to the primary writer protein Mettl1, while the precise roles of reader and eraser proteins in skeletal development remain to be elucidated in future studies.

## Conclusion

**Figure 9:**
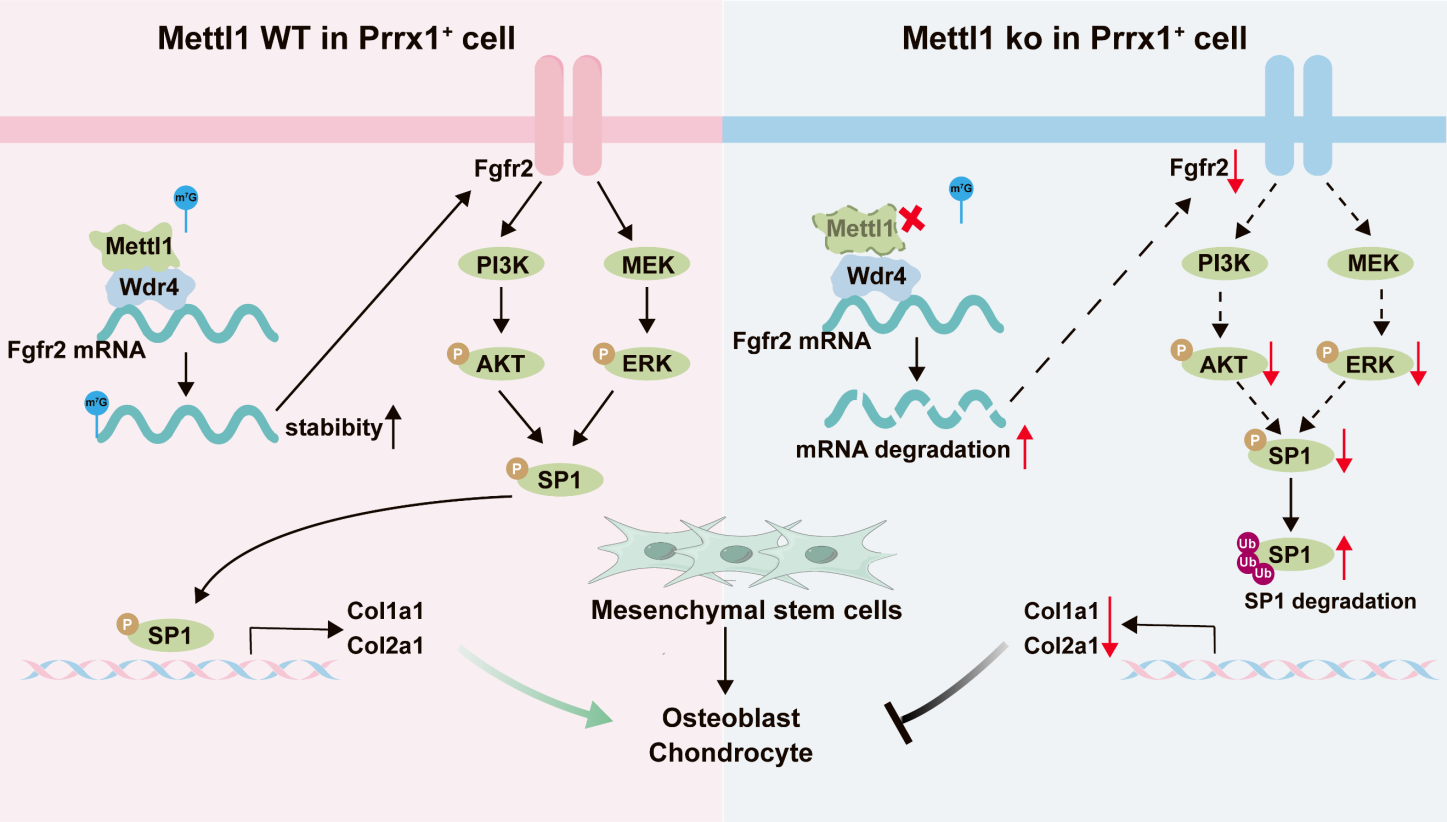
** The working model depicting the overall findings of this study**.

In conclusion, this study demonstrates that Mettl1-mediated m^7^G modifications are integral to osteogenic and chondrogenic differentiation of mesenchymal stem cells during skeletal development, via the Fgfr2-Sp1-Col1a1/Col2a1 axis (Figure 9). Overall, this study not only advances our understanding of RNA modifications in skeletal development but also provides a foundation for developing innovative therapies for skeletal diseases.

## Supplementary Material

Supplementary figures.

## Abbreviations

MSCs: Mesenchymal stem cells
m^7^G: N^7^-methylguanosine
cko: conditional knockout
WT: Wild type
Mut: Mutant
scRNA-seq: single-cell RNA sequencing
CHX: Cycloheximide
