# Supplementary material for: Mettl1-mediated m7G modification of Fgfr2 regulates osteogenic and chondrogenic differentiation of mesenchymal stem cells
Source: Int J Biol Sci. 2025 Sep 3;21(13):5704–24. doi: 10.7150/ijbs.114889 (PMC12509694; doi:10.7150/ijbs.114889)

Supplementary Figure S1. ***Mettl1***$^{flox/flox}$***Prrx1***^{cre} **mice exhibit skeletal dysplasia phenotype.** (A) The DNA gel electrophoresis assay to determine the genotypes of the indicated mice. (B) Expression levels of Mettl1 mRNA were measured in femurs and tibias of Control and cko mice at P3 by qPCR (n=3). (C) Western blot analysis showing Mettl1 protein levels in femurs and tibias of Control and cko mice at P3. (D) Alcian blue-alizarin red S staining of the spine and tailbone. Scale bar = 5mm. (E) Representative micro-CT images of the roof of skull from Control and cko mice at P3, P7, P14 and P25. (F) Representative images of organs and tissues from Control and cko mice at P25. (G-J) Quantification of positive cells in the immunostaining analysis shown in Figure 2D-G (n = 3 per group). (K) Quantification of thickness data shown in Figure 2J (n = 3 per group). The statistical data are represented as the means ± SDs, ****P* < 0.005, *****P* < 0.001, ns = no significant difference.

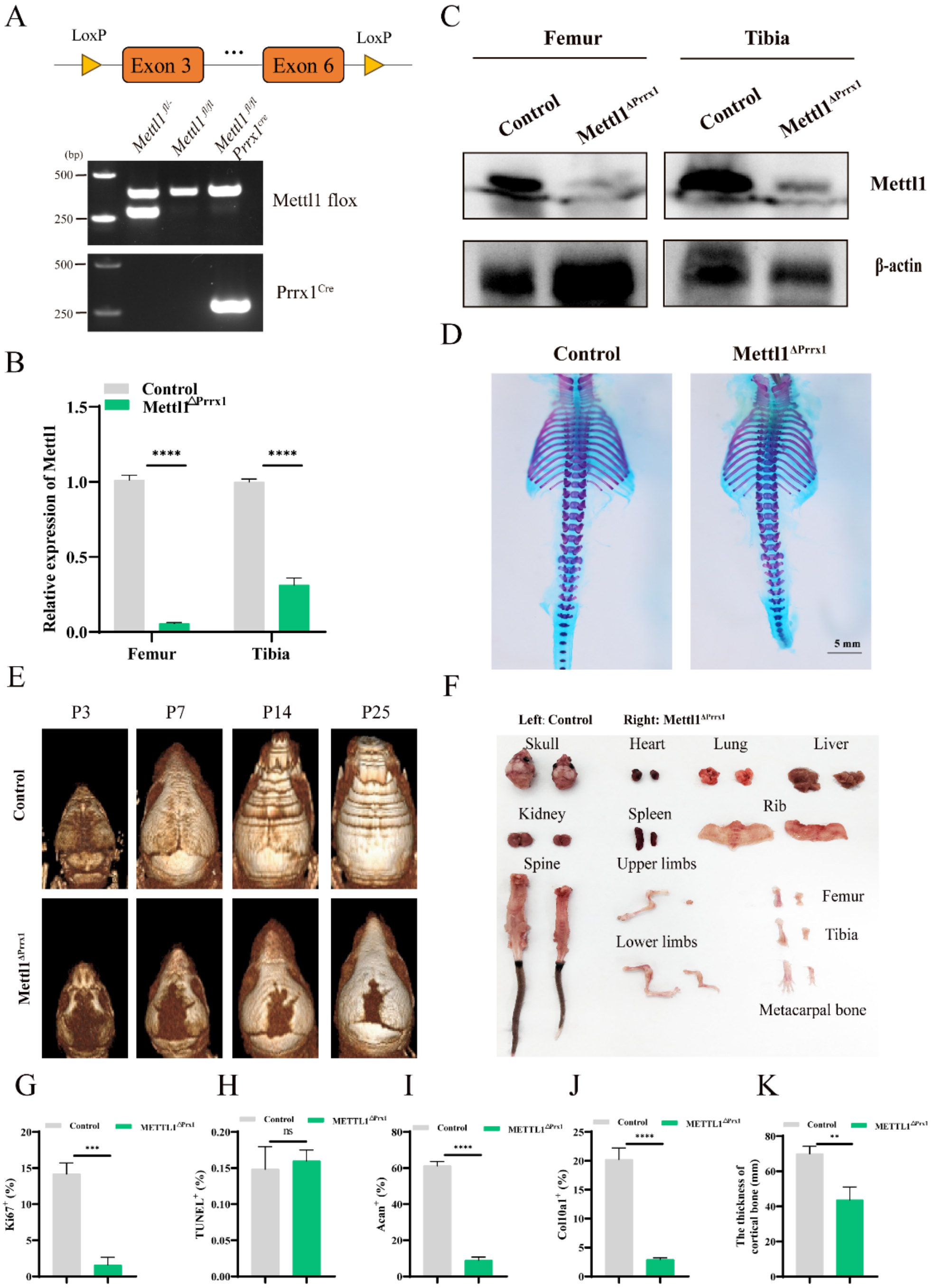

Supplementary Figure S2. Single-cell RNA-sequencing analysis of cell mixture of mouse femurs. (A-D, left) UMAP projection of integrated scRNA-seq data, labeled by corresponding cell type, containing 8,440 (P3), 15,051 (P7), 16,523 (P14) and 12,716 (P25) cells. (A-D, right) Dot plot of typical gene expression profiles of the identified cell clusters.

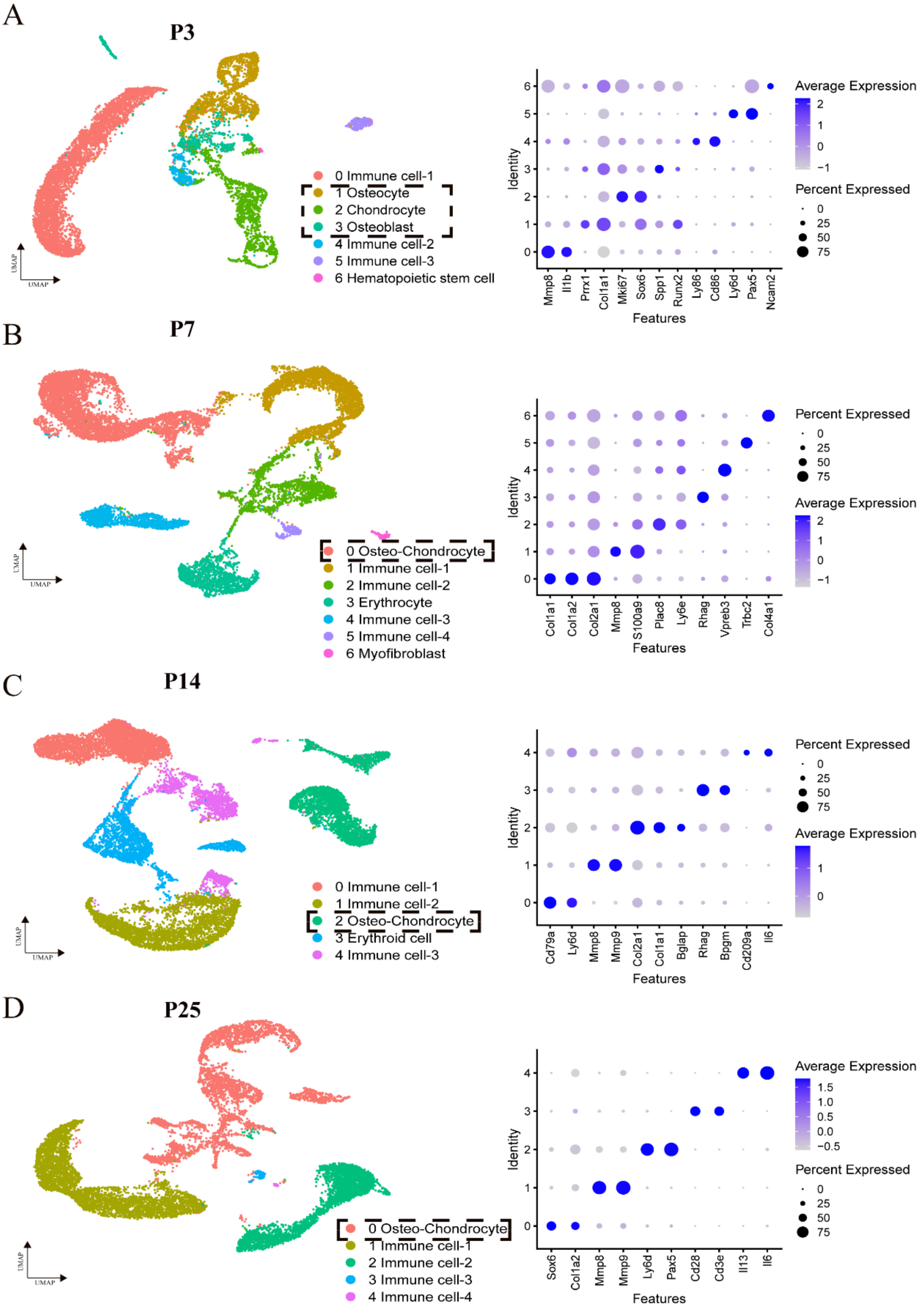

Supplementary Figure S3. Single-cell RNA sequencing analysis reveals alterations in mesenchymal lineages. (A) Pseudotime trajectory of chondrocytes and osteoblasts with distinct color coding for each group at different time points. (B) Pseudotime trajectory of chondrocytes and osteoblasts with distinct color coding for each cluster identified in Figure 3D. (C) Pie charts illustrating the distribution of the two groups across each pseudotime state at different time points. (D) The lollipop chart depicting distinct GO enrichment analysis of 523 downregulated genes in chondrocytes. (E) The lollipop chart depicting distinct GO enrichment analysis of 742 downregulated genes in osteoblasts.

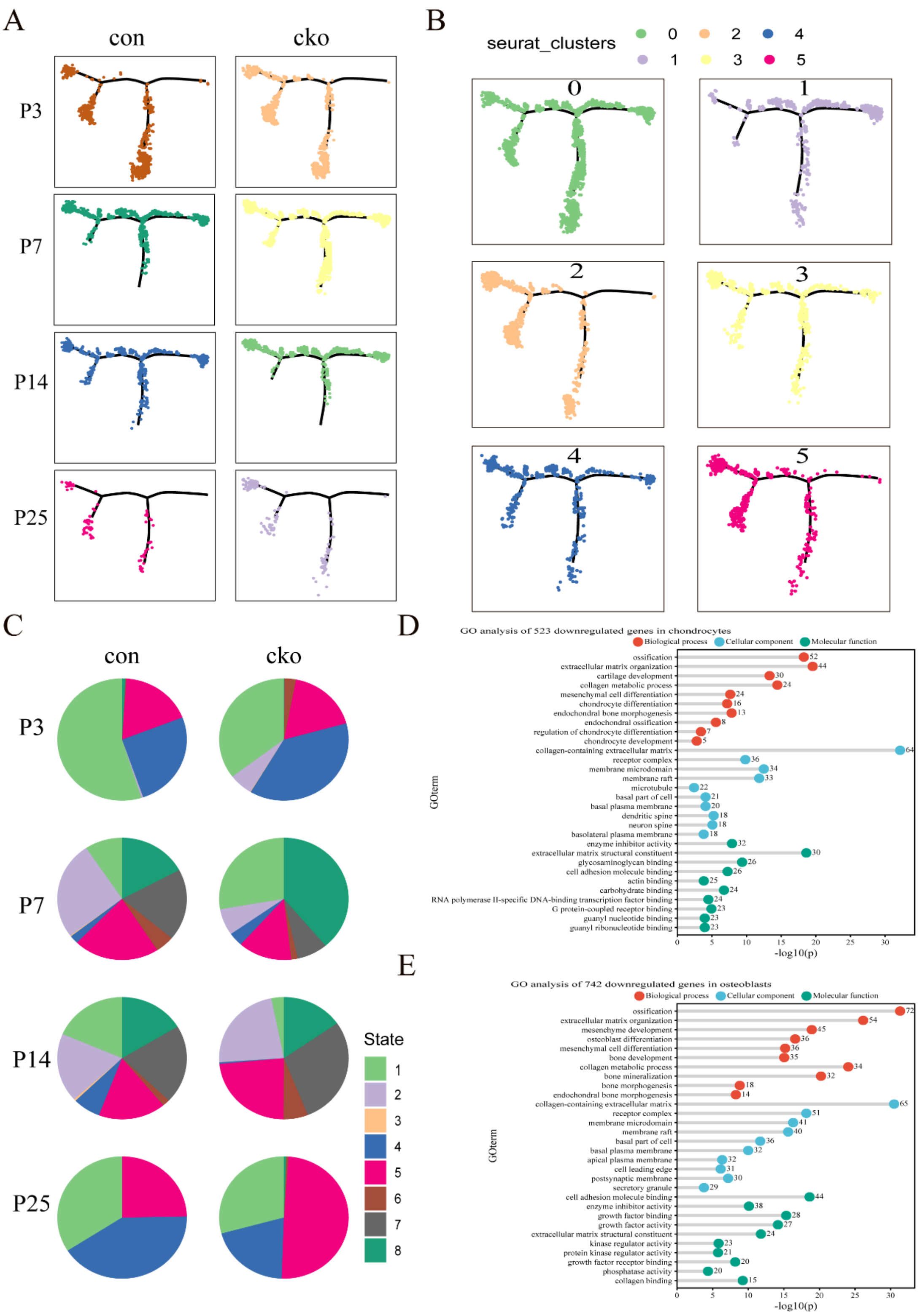
A
con
cko
P3
P7
P14
P25
B
seurat_clusters
0
1
2
3
4
5
C
con
cko
P3
P7
P14
P25
State
1
2
3
4
5
6
7
8
D
GO analysis of 523 downregulated genes in chondrocytes
Biological process
Cellular component
Molecular function
GOterm
ossification 52
extracellular matrix organization 44
cartilage development 30
collagen metabolic process 24
mesenchymal cell differentiation 24
chondrocyte differentiation 16
endochondral bone morphogenesis 13
endochondral ossification 8
regulation of chondrocyte differentiation 7
chondrocyte development 5
collagen-containing extracellular matrix 64
receptor complex 36
membrane microdomain 34
membrane raft 33
microtubule 22
basal part of cell 21
basal plasma membrane 20
dendritic spine 18
neuron spine 18
basolateral plasma membrane 18
enzyme inhibitor activity 32
extracellular matrix structural constituent 30
glycosaminoglycan binding 26
cell adhesion molecule binding 26
actin binding 25
carbohydrate binding 24
RNA polymerase II-specific DNA-binding transcription factor binding 24
G protein-coupled receptor binding 23
guanyl nucleotide binding 23
guanyl ribonucleotide binding 23
0 5 10 15 20 25 30
-log10(p)
E
GO analysis of 742 downregulated genes in osteoblasts
Biological process
Cellular component
Molecular function
GOterm
ossification 72
extracellular matrix organization 54
mesenchyme development 45
osteoblast differentiation 36
mesenchymal cell differentiation 36
bone development 35
collagen metabolic process 34
bone mineralization 32
bone morphogenesis 18
endochondral bone morphogenesis 14
collagen-containing extracellular matrix 65
receptor complex 51
membrane microdomain 41
membrane raft 40
basal part of cell 36
basal plasma membrane 32
apical plasma membrane 32
cell leading edge 31
postsynaptic membrane 30
secretory granule 29
cell adhesion molecule binding 44
enzyme inhibitor activity 38
growth factor binding 28
growth factor activity 27
extracellular matrix structural constituent 24
kinase regulator activity 23
protein kinase regulator activity 21
growth factor receptor binding 20
phosphatase activity 20
collagen binding 15
0 5 10 15 20 25 30
-log10(p)

Supplementary Figure S4. The expression levels of Runx2 and Runx3 following Mettl1 knockout. (A) Western blot analysis showing Runx2 protein levels in femurs and tibias of Control and cko mice at P3 (left). The protein levels of Runx2 in primary mice MSCs transfected with the indicated constructs (middle). Western blot analysis of Runx2 in control group and Mettl1 ko group with or without SEW2871 (0.5 μM/ml) (right). (B) Western blot analysis showing Runx3 protein levels in femurs and tibias of Control and cko mice at P3 (left). The protein levels of Runx3 in primary mice MSCs transfected with the indicated constructs (middle). Western blot analysis of Runx3 in control group and Mettl1 ko group with or without SEW2871 (0.5 μM/ml) (right).

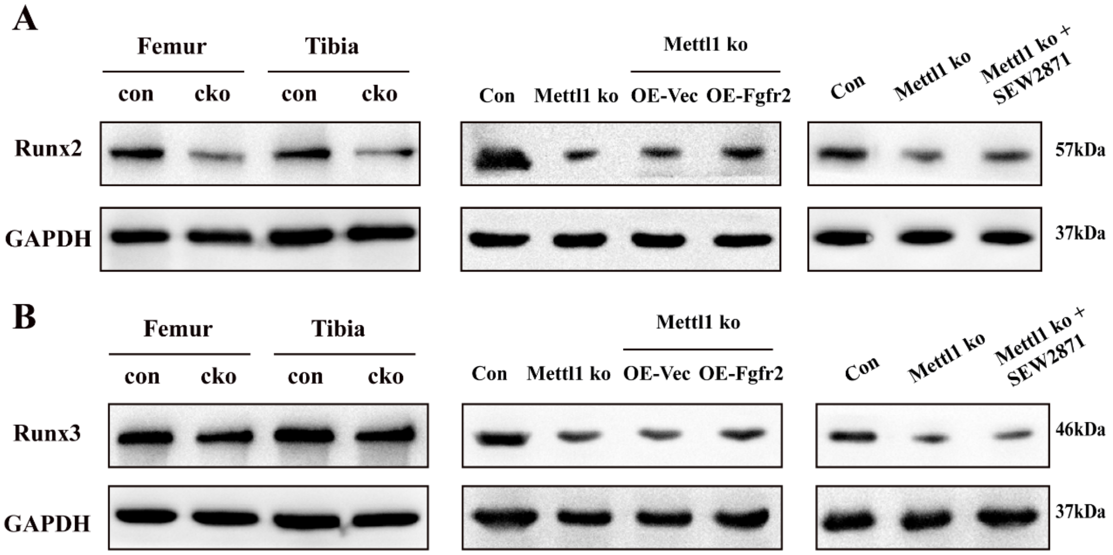

Supplement: Supplementary file 1 — Supplementary figures. [file ijbsv21p5704s1.pdf]
